# Evaluation of 3D Printed Soft Robots in Radiation Environments and Comparison With Molded Counterparts

**DOI:** 10.3389/frobt.2019.00040

**Published:** 2019-05-24

**Authors:** Osman Dogan Yirmibeşoğlu, Tyler Oshiro, Gina Olson, Camille Palmer, Yigit Mengüç

**Affiliations:** ^1^Collaborative Robotics and Intelligent Systems Institute, Oregon State University, Corvallis, OR, United States; ^2^School of Nuclear Science and Engineering, Oregon State University, Corvallis, OR, United States

**Keywords:** 3D printing, additive manufacturing, soft robotics, radiation environments, soft actuators, nuclear robotics, silicone elastomer

## Abstract

Robots have an important role during inspection, clean-up, and sample collection in unstructured radiation environments inaccessible to humans. The advantages of soft robots, such as body morphing, high compliance, and energy absorption during impact, make them suitable for operating under extreme conditions. Despite their promise, the usefulness of soft robots under a radiation environment has yet to be assessed. In this work, we evaluate the effectiveness of soft robots fabricated from polydimethylsiloxane (PDMS), a common fabrication material, under radiation for the first time. We investigated gamma-induced mechanical damage in the PDMS materials' mechanical properties, including elongation, tensile strength, and stiffness. We selected three radiation environments from the nuclear industry to represent a wide range of radiation and then submerged a 3D printed hexapus robot into a radiation environment to estimate its operation time. Finally, to test the reliability of the 3D printed soft robots, we compared their performances with molded counterparts. To analyze performance results in detail, we also investigated dimensional errors and the effects of fabrication methods, nozzle size, and print direction on the stiffness of PDMS material. Results of this study show that with increasing exposure to gamma irradiation, the mechanical properties of PDMS decrease in functionality but are minimally impacted up to 20 kGy gamma radiation. Considering the fractional changes to the PDMS mechanical properties, it is safe to assume that soft robots could operate for 12 h in two of the three proposed radiation environments. We also verified that the 3D printed soft robots can perform better than or equal to their molded counterparts while being more reliable.

## Introduction

Robotics research has a significant role when utilizing robots for inspection, clean-up, and sample collection in hazardous environments inaccessible to humans. Especially when it comes to radiation environments, their deployment minimizes unnecessary exposure of workers to the harmful effects of radiation (Moore, 1985). Accordingly, robots have a long history in the nuclear field, from the incident at Three Mile Island, Reactor 2 (TMI-2) in 1979 to the 2011 disaster at Fukushima Daiichi Nuclear Power Plant (Urabe and Stapczynski, 2017). At TMI-2, robots performed surveillance, inspection, and decontamination tasks following the meltdown (Hess and Metzger, 1985; Lovering, 2009). Soon after the nuclear power plant accident in Fukushima, Japan, the existing Quince robot was modified to perform inspection and sampling tasks in two of the affected units (Nagatani et al., 2011a). This robot completed several objectives before becoming irretrievably lost (Nagatani et al., 2011b). Recently, equipment specifically designed to operate within Fukushima, including Toshiba's Scorpion and Sunfish models, have been introduced to perform additional surveillance (Fackler, 2017). However, most of the deployed robots faced a similar problem: getting stuck or tangled in debris. They also suffered from circuit malfunctions due to high doses of radiation, especially if hardened parts were not used in fabrication (Fackler, 2017). The contaminated and malfunctioning robots were abandoned inside the reactor, at a total loss of the equipment's capital cost (Mary-Ann, 2015; Sheldrick and Funakoshi, 2016; McCurry, 2017). These circumstances raised a central question: Can we use low-cost soft robots in radiation environments? Soft robots provide advantages over rigid robots in terms of body morphing (Laschi et al., 2016), absorbing the energy of an impact or collision (Lee et al., 2017), high compliance (Rus and Tolley, 2015), and cheaper fabrication costs (Hill et al., 2000). The most significant of these advantages is the robot's ability to conform to different obstacles and terrains in various radiation environments, especially during passage through non-traditional entryways when doors and access points are blocked. Moreover, millimetric (Hu et al., 2018; Ranzani et al., 2018) scale soft robots may also provide considerable advantages under radiation environments.

In order to evaluate the effectiveness of soft robots under radiation, the convenience of the fabrication material for the environment plays a crucial role. The earliest investigations of the effect of gamma radiation on polydimethylsiloxane (PDMS) were performed by Charlesby (Charlesby, 1955) and Miller (Miller, 1960) in the late 1950s. They determined that the degree of crosslinking induced by radiation is a function of dose and demonstrates a direct-response relationship. Charlesby calculated a 32-eV energy absorption requirement per crosslink and Miller calculated a crosslinking yield of 3.0% for irradiation by electrons. Notably, both studies were performed on the liquid form of PDMS rather than the cured form considered in soft robotic applications. Therefore, to fill this research gap, and to estimate fabricated soft robots' operation time under radiation, we investigated gamma-induced mechanical damage in PDMS and sent a 3D printed soft robot into an underwater radiation environment. The main reasons for selecting the 3D printing method over molding to fabricate soft robots will be detailed in the following paragraphs.

To send a soft robot in an unstructured radiation environment for inspection purposes or delivery tasks, it must offer significant dexterity and mechanical compliance, with minimum control requirements. However, disadvantages of soft robots such as limited afforded strength and payload (Lee et al., 2017), limited control and autonomy (Trivedi et al., 2008; Singh and Krishna, 2014), need for tethering (Majidi, 2014; Schmitt et al., 2018), and limited sensory equipment (Rus and Tolley, 2015; Lee et al., 2017) still need to be overcome. To meet some of these demands, the soft actuators within the soft robot must enhance their functionality, which is limited by fabrication techniques (Marchese et al., 2015). Since conventional soft robot manufacturing techniques such as lamination casting (also known as soft lithography) (Xia and Whitesides, 1998; Tolley et al., 2014), retractable pin casting (Marchese et al., 2014, 2015), lost wax casting (Sias, 2005; Marchese et al., 2015), and rotomolding (Zhao et al., 2015) restrict possible geometries, shapes, complexity, and scale of the manufactured soft robots, we choose to focus on additive manufacturing (AM) methods (Truby and Lewis, 2016; Walker et al., 2019). However, the most commonly used AM techniques, such as stereolithography (SLA) (Peele et al., 2015), fused filament fabrication (FFF) (Yap et al., 2016), and PolyJet (Drotman et al., 2017) are not suitable for 3D printing PDMS material for fabricating soft robots (Trimmer et al., 2015; Laschi et al., 2016; Kastor et al., 2017).

One of the first examples of a 3D printed soft actuator (Peele et al., 2015) failed at around 40% strain (after approximately nine cycles) due to photopolymer SLA materials while their molded counterparts fabricated with the PDMS materials were able to undergo more than 600% strain (Mosadegh et al., 2014). Alternatively, another 3D printed soft actuator manufactured through FFF methods was limited to the NinjaFlex (NinjaTek, PA) thermoplastic material with a Shore hardness of 85A (Yap et al., 2016). More recent 3D printed soft robots were manufactured with PolyJet technology which allowed researchers to (1) manufacture a quadrupedal robot with soft legs capable of two axis rotation (Drotman et al., 2017), (2) create a material stiffness gradient within the soft robot body (Bartlett et al., 2015), and (3) 3D co-print solids (flexible, rigid) and liquids to fabricate hydraulically actuated components (MacCurdy et al., 2016). However, the commercially available materials (Stratasys, MN) used in this process were limited by Shore hardness (ranging between 27A and 95A).

To overcome strain limitations and use PDMS materials within AM, researchers focused on direct ink writing (DIW) techniques. Ober et al. analyzed the behavior of complex fluids and developed a micro-scale active mixing system for two-part materials, and successfully 3D printed PDMS objects (Hardin et al., 2015; Ober et al., 2015), but they did not demonstrate the fabrication of soft actuators or robots. Instead of using two-part PDMS materials, Plott et al. used moisture-cured silicone elastomer to successfully 3D print finger pneumatic actuators (Plott and Shih, 2017). Unfortunately, their printing technique restricted the achievable geometry as it required near voidless construction.

**Figure 1:**
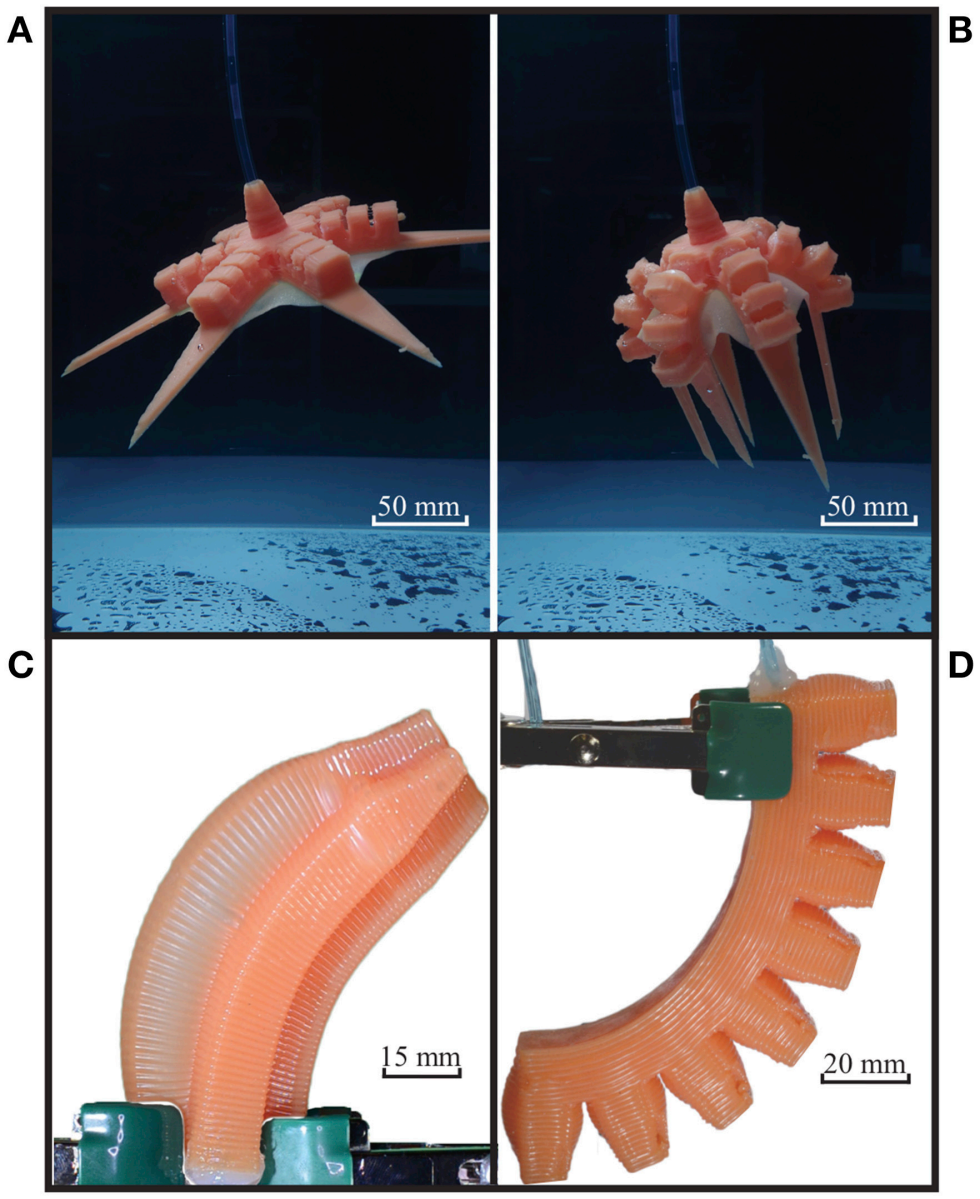
3D printed soft robots. **(A)** Hexapus nominal state. **(B)** Hexapus actuated state. **(C)** 4 channel tentacle (Yirmibesoglu et al., 2018). **(D)** Pneu-net actuator (Yirmibesoglu et al., 2018).

Considering the limitations of current state-of-the-art PDMS printing, the soft robotics community has yet to match the performance of the molded functional soft robots made from PDMS materials with 3D printing technology. In prior work (Yirmibesoglu et al., 2018), we developed a 3D printer to address this research gap. Here by modifying the previous printer design, we improved the complexity and increased the scale of the fabricated soft robots, which enabled us to 3D print a hexapus robot (Figures 1A,B) for testing soft robots under a radiation environment.

In this work, in order to assess the usefulness of soft robotics under radiation, we selected 3 radiation environments to provide a wide scope of operation. To estimate fabricated soft robots' operation time in these radiation environments, we measured gamma-induced changes in mechanical properties such as elongation, tensile strength, and compression of the PDMS material. The viability of the soft robots under 3 selected radiation environments was analyzed based on PDMS behavior after irradiation. Later, a 3D printed soft hexapus robot (Frame et al., 2018) was operated in a radiation environment, and its absorbed dose rate was measured to estimate its operation time. By using 3D printing as the fabrication method, we increased design complexity of the hexapus robot, which enabled us to test its operation time under radiation environment. Finally, to ensure the reliability of the 3D printed soft robots we investigated the effects of fabrication methods, nozzle size, and print direction on the stiffness of the PDMS material.

This paper is organized as follows. In section 3D printing of silicone material, we introduced the improvements to the 3D silicone printer that enabled us to 3D print a hexapus robot. In section Materials and methods, we detailed the protocols to measure the effect of gamma irradiation on PDMS material and select radiation environments. Also, experimental methods and setups for radiation experiments and robot performance comparisons are detailed. In section Results and discussion, we analyzed the changes in the mechanical properties of PDMS samples after gamma irradiation and we measured the gamma irradiation absorbed by the 3D printed hexapus robot submerged into bulk shield tank while repeating pull and push motions. After that, we conducted blocked force and bend angle experiments to compare the performance differences between our 3D printed soft robots (4 channel tentacle Figure 1C and Pneu-net actuator Figure 1D) and their molded counterparts. We also measured the effects of fabrication methods, nozzle sizes, and print directions on the stiffness of the fabricated PDMS material. Finally, in section Conclusion, conclusions and future work are presented. Authors also provided a table of the acronyms (Supplementary Table 1) used throughout the paper to help readers.

## 3D Printing of Silicone Material

**Figure 2:**
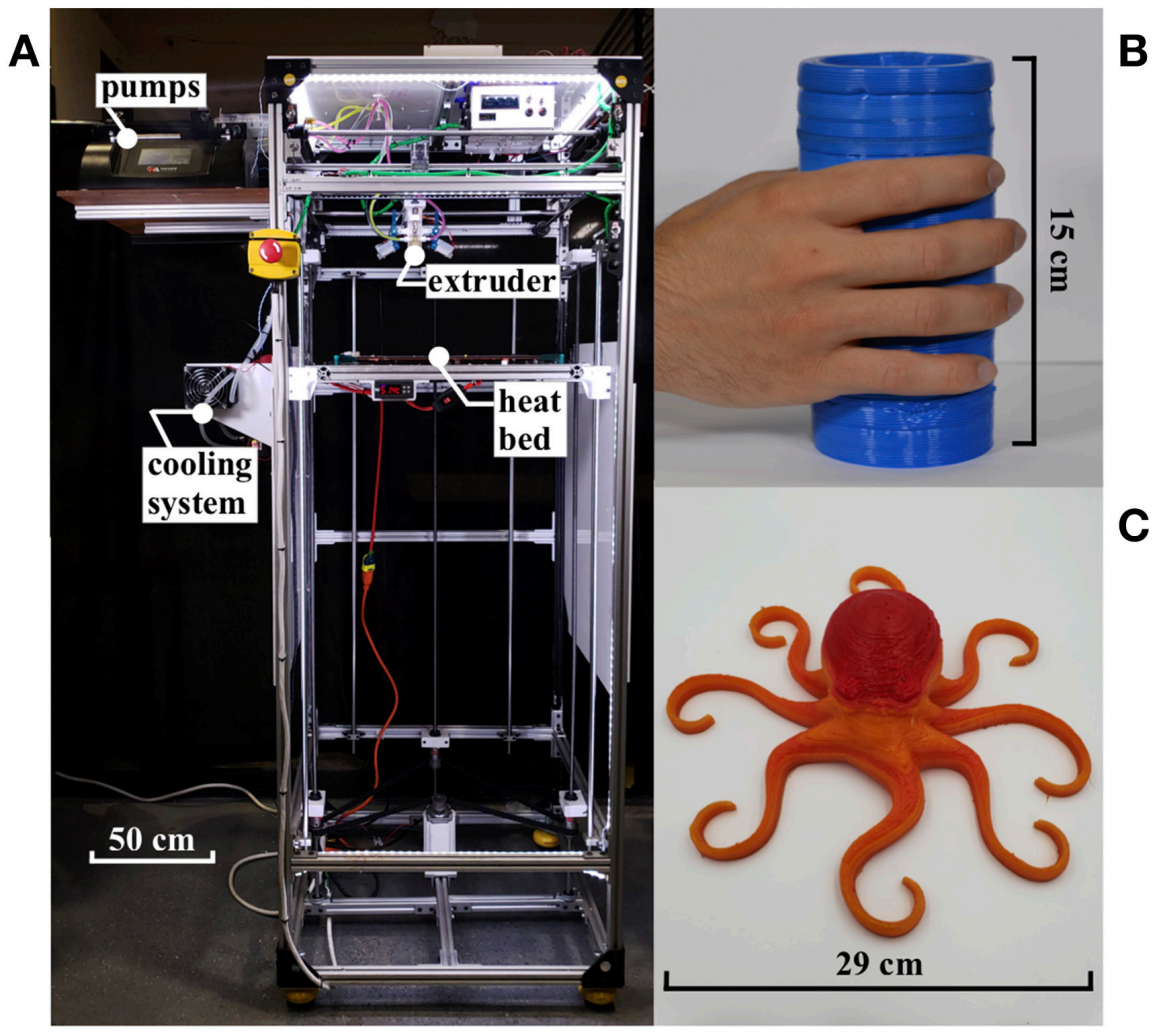
**(A)** 3D silicone printer. **(B)** 15 cm tall cylinder, 3D printed in 5 h without collapsing. **(C)** Soft octopus sculpture (0.79 kg), 3D printed in 18 h.

In this section, we describe improvements to the previous 3D silicone printer design that enabled us to 3D print a hexapus robot (Figure 1A) capable of swimming in an underwater radiation environment. For additional instrument design details, please refer to the original paper (Yirmibesoglu et al., 2018). The major changes in the 3D printer setup (Figure 2A) include new material, modified extruder, and new print parameters. A detailed explanation of the 3D printer including a preliminary benchmark study for AM of soft materials is provided in the Supplementary Material.

### Print Material

The print material we used was Dragon Skin 10 (DS10) very fast, a two-part platinum cure silicone (Smooth-On, PA), in combination with 1 wt % Thi-Vex (Smooth-On, PA), a viscosifying agent used to thicken the formulation to improve print fidelity based on our previous paper (Yirmibesoglu et al., 2018). However, due to high loads on the syringe pumps at 75 cm tube length (Supplementary Figure 1), we added 10 wt % silicone thinner (Smooth-On, PA) into the formula, based on the findings of a recent study (Walker et al., 2018). With the improved formula, the accumulated load decreased from 351 N down to 252 N. The high viscosity print material also prevented bubble formation at the macro level.

### Extruder Mechanism

**Figure 3:**
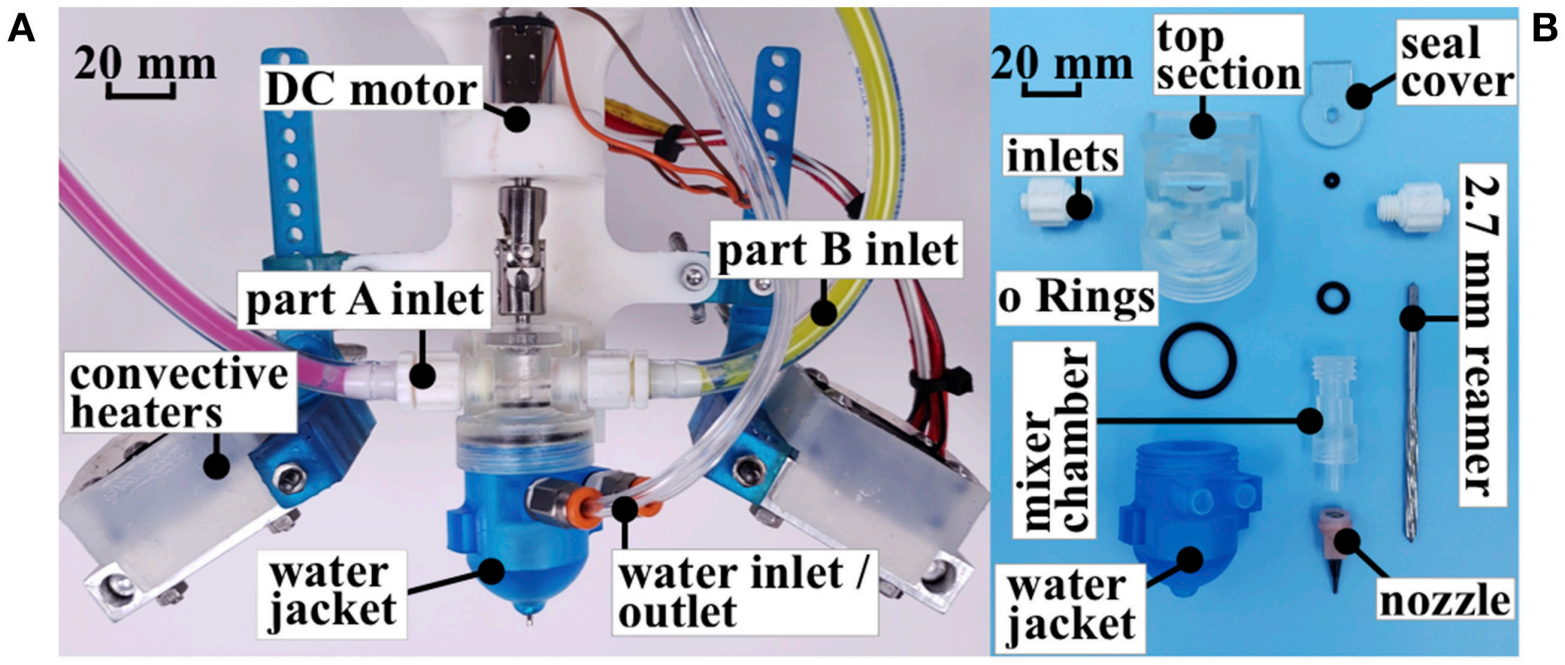
**(A)** Extruder mechanism with water jacket addition. **(B)** Mixer assembly.

We improved the extruder mechanism from previous works (Ober et al., 2015; Morrow et al., 2017; Yirmibesoglu et al., 2018) in several ways to print taller soft objects (Figure 2B) and to achieve extended print times (Figure 2C). First, we decreased the mixer chamber volume. In the old design, the cross section diameter of the mixer chamber was 10.8 mm, resulting in 8.1 times the volume to be initially filled by the mixed material before extrusion, compared to the new mixer chamber design. With a bigger volume, the amount of the time that the material mix stayed inside the mixer was longer. Since the heat bed (Figure 2A) and convective heating (Figure 3A) created a hot environment around the mixer chamber, this resulted in an increase in the crosslinking rate of the mixed material. With higher crosslinking, the material became more viscous and was unable to pass through smaller nozzle sizes resulting in a clogged mixer. The smaller chamber volume decreased the amount of time required to discharge the mixed material from the mixer chamber before the crosslinking turns the material into a highly viscous state.

Second, by adding the water jacket system (Figure 3B), we circulated cold water around the mixer chamber using a commercially available pump (Water cooling kit, E3D-online, UK) to maintain the temperature of the mixed material below 25°C while the surrounding environment was between 45° and 80°C. The combination of the smaller volume mixer chamber and water jacket systems, kept the crosslinking rate of the mixed material low enough, so that discharge of the mixed material completed before the mixer got clogged resulting in extended print times. Our longest print took 18 h using a 0.839 mm nozzle and weighed 0.79 kg (Figure 2C). Achieving extended print times was the key to fabricate the hexapus robot for radiation tests, which was 3D printed in 11 h with DS10-fast composition. The step-by-step guide to manufacturing the initial version of this extruder mechanism is publicly available on the Soft Robotics Toolkit^1^.

### Print Parameters and Limitations

After modifying the extruder to print taller silicone objects and extend print times, to achieve high resolution with prints we followed a recent strategy (Yuk and Zhao, 2018) that benefits from the deformation of viscoelastic inks. With the guidance of this study, by mainly tuning print parameters such as print speed, flow rate, and layer height we deposited lines between die-swelling, equi-dimensional, and thinning print modes (Yuk and Zhao, 2018). The transition between the modes was achieved by changing the print speed or flow rate. Our latest list of parameters for a successful print is available in Supplementary Table 3. Moreover, there are a couple of design limitations observed in robot fabrication. A list of design limitations with the recommended parameters is available in Supplementary Table 4. By considering the printer modifications, improved print parameters and design limitations detailed in this section, we achieved the AM of the hexapus robot.

## Materials and Methods

In this section, we first detail the protocols to measure the effect of gamma irradiation on the mechanical properties of the PDMS material. We then describe the selection of radiation environments for soft robot operation and how to measure the irradiation dose of the hexapus robot. Second, we describe the comparison methods for measuring performance and ensuring the reliability of the 3D printed soft robots compared to their molded counterparts. Finally, we describe experimental methods to identify the causes of performance differences between 3D printed and molded soft robots.

### Mechanical Testing of Gamma-Induced PDMS Samples

In order to evaluate soft robot reliability in gamma radiation environments, samples of PDMS were irradiated in a GammaCell 220 to a high dose rate from a Co-60 source (a radioactive isotope of the element cobalt). We prepared 27 dumbbell test pieces and 20 disc-shaped compression samples were prepared from DS10-fast silicone. The sample dimensions were 29.0 mm in diameter and 12.5 mm in thickness. To create samples of both kinds, equal parts by weight of DS10-fast part A and B were mixed and poured into molds and placed under −100 kPa vacuum for 5 min. Then these samples were placed in a 60°C oven for 15 min and were allowed to rest to reach the final mechanical properties. Finally, samples were irradiated at six increments of gamma-only doses from 7 to 400 kGy, with at least three samples tested at each cumulative dose. A total of 6 samples were reserved as a control group.

**Figure 4:**
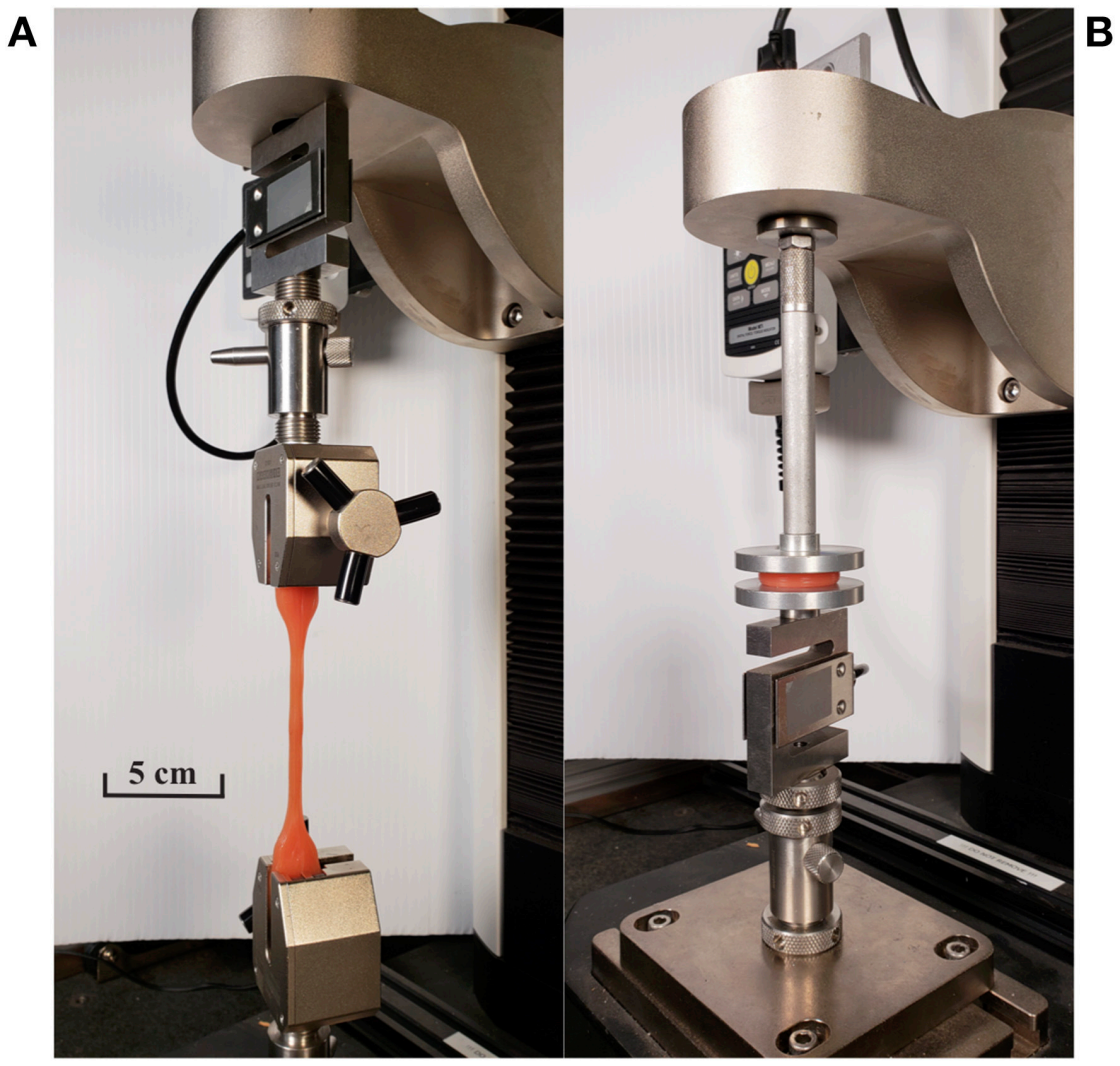
Testing irradiated dumbbell and disc samples with Mark-10 motorized test stand (Oshiro, 2018). **(A)** Tensile strength and elongation testing setups. **(B)** Compression testing setup.

After gamma irradiations were completed, samples were subjected to mechanical tests in a motorized tension/compression stand (ESM1500, Mark-10, NY) (Figures 4A,B). For the tensile tests, the length of the narrow portion of the sample (L1), was increased by separating the sample ends at a rate of 250 mm/min. The samples were stretched until failure while measuring L1 and the tensile force to determine the “tensile strength at break” and “elongation at break” according to ASTM D412-16. For compression testing, the height of the sample was decreased at a rate of 50 mm/min to 75% of the original value, then released.

### Selecting Radiation Environments for Soft Robot Operation

To provide context for this assessment, three radiation environments were considered to represent the general diversity of potential applications. Environments in the nuclear power industry are used because they are well-characterized and encompass real-life scenarios of various dose rates. The three radiation environments are selected from a guide (Sharp and Garlick, 1994): (1) Spent fuel storage pools, (2) Vitrified waste and the vitrification process, and (3) Deactivation of a generic pressurized water reactor (PWR). Available dose rate ranges and averages from these documented environments were used to calculate a cumulative dose assuming as 12 h robot task time. In all cases, the most conservative (highest documented dose rate) was assumed, which resulted in a 12 h cumulative dose of 120 kGy for spent fuel storage pools, 21.6 kGy for vitrified waste and the vitrification process, and 12 kGy for deactivation of a generic PWR. The cumulative dose, rather than the dose rate, is applicable here because past research on PDMS (Comstock, 1989) suggests that radiation is a function of cumulative dose and is not heavily dependent on dose rate.

### Measuring the Irradiation Dose of the Hexapus Robot in the Bulk Shield Tank

To estimate the 3D printed soft robots' operation time we submerged the hexapus robot into a radioactive bulk shield tank. The hexapus robot was 46 mm tall and 286 mm in diameter. Six actuation arms consisted of Pneu-net structures were place 60° apart from each other. Tap water was pumped and withdrawn in consecutive cycles into the hexapus by using a 60 ml syringe attached to a syringe pump (NE-4000, New Era, NY) at the max pump speed of 95.99 ml/min. We switched from pneumatic to hydraulic to avoid the hexapus robot floating on the surface. The hexapus and the pump were connected by 6 meters of soft tubing with an inner diameter of 3.2 mm (Supplementary Figure 3). Later, the hexapus was submerged into a radioactive bulk shield tank (2.7 × 2.4 × 3.7 m–width x height × depth), (Supplementary Video 1). The tank was under the effect of two main gamma irradiation sources: (1) a used graphite reflector, and (2) radiation flux coming from the neighboring operating nuclear reactor. An underwater ion chamber (CPMU, Technical Associates, CA) was used to measure the dose rate next to the hexapus (Supplementary Figure 4). In addition, we were unable to measure the performance change of the hexapus' pull and push motions with absorbed dose rate (Supplementary Video 1) as our underwater camera equipment was not suitable for radiation environments.

### Measuring the Performance of the 3D Printed and Molded Soft Robots

Reliable operation of a soft robot is a vital step before evaluating the effectiveness of soft robots under radiation. To ensure the reliability of the 3D printed soft robots and verify their performances we compared them with the molded counterparts. However, we did not fabricate a molded version of the hexapus robot because of the laborious and time intensive (up to 3 days) manufacturing steps which are beyond the scope of the current work. Instead, we fabricated 4 channel tentacles and Pneu-net actuators that are part of the hexapus actuation design.

The blocked force and bend angle experiments were used to measure the performance of each robot in response to given pressure (Holland et al., 2014). Fabricated 4 channel tentacles (Marchese and Rus, 2016) were used for identifying the effects of 3D printing compared to lost wax casting on robots' performance. Next, fabricated Pneu-net actuators (Ilievski et al., 2011; Mosadegh et al., 2014) were used for identifying the effects of 3D printing compared to lamination casting on robots' performance. We selected our robot designs based on the most common (excluding the use of fabric) bending principles: eccentric void asymmetry (4 channel tentacle) and corrugated membrane asymmetry (Pneu-net actuator, hexapus) (Gorissen et al., 2017). Differences between fabrication steps of the tested robots are provided in Supplementary Table 5, and the experimental setup used for the performance comparison tests can be seen in Supplementary Figure 5.

**Figure 5:**
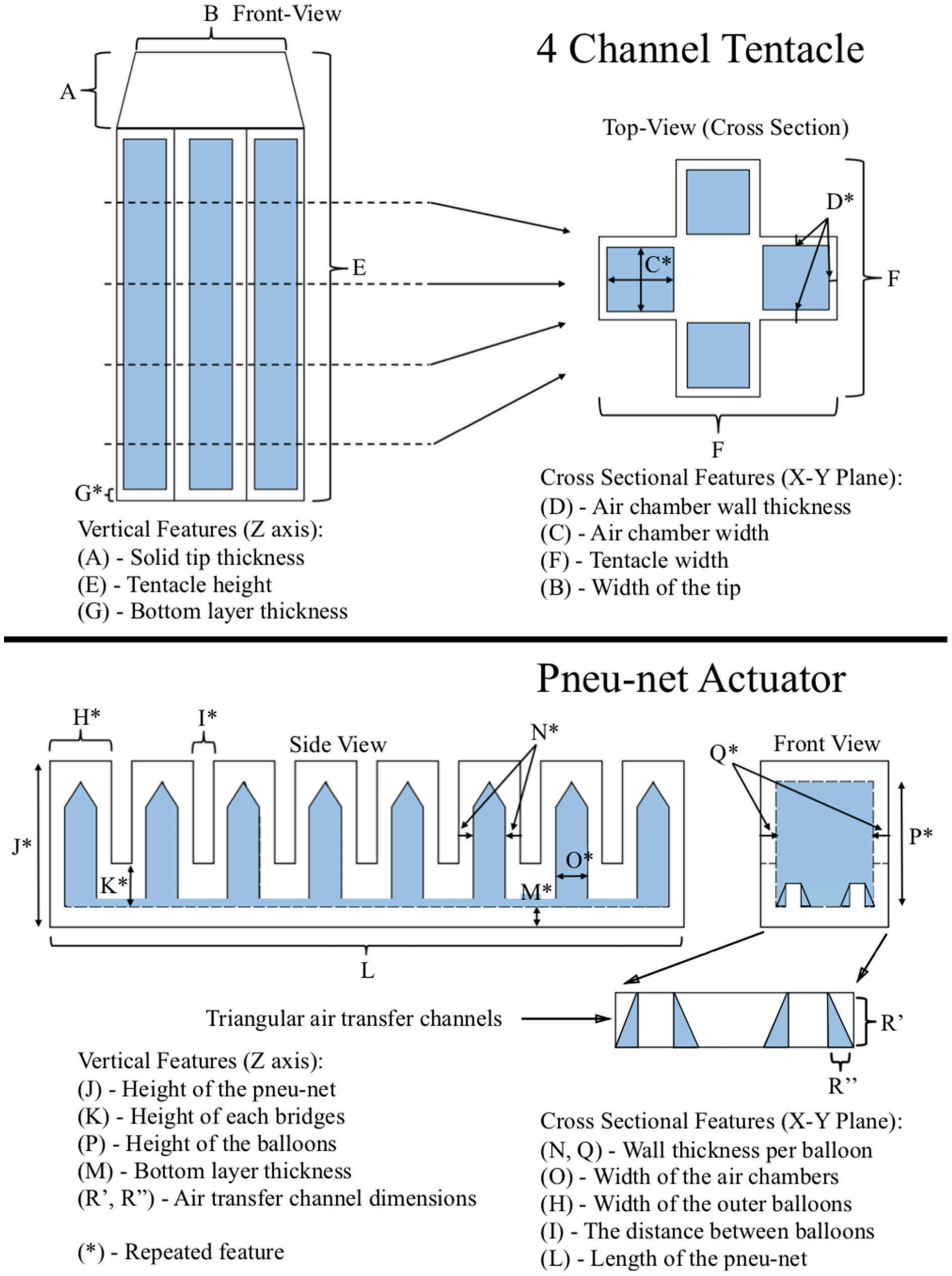
Measured feature descriptions: 4 channel tentacle (top), Pneu-net actuator (bottom) (Yirmibesoglu et al., 2018).

In order to understand any performance differences in the blocked force and bend experiments, we initially calculated dimensional errors. Features measured and compared against CAD models were shown in Figure 5 for both the 4-channel tentacle [Top] and Pneu-net actuator [Bottom]. The cross-sectional features (those in the X-Y plane) and the vertical features (those in the Z-axis) were measured. We consolidated features together by averaging their mean values. Each variable was measured at four random locations on the soft robots with a digital caliper and percent error deviations calculated.

### Measuring the Effects of Fabrication Methods, Nozzle Size, and Print Direction

**Figure 6:**
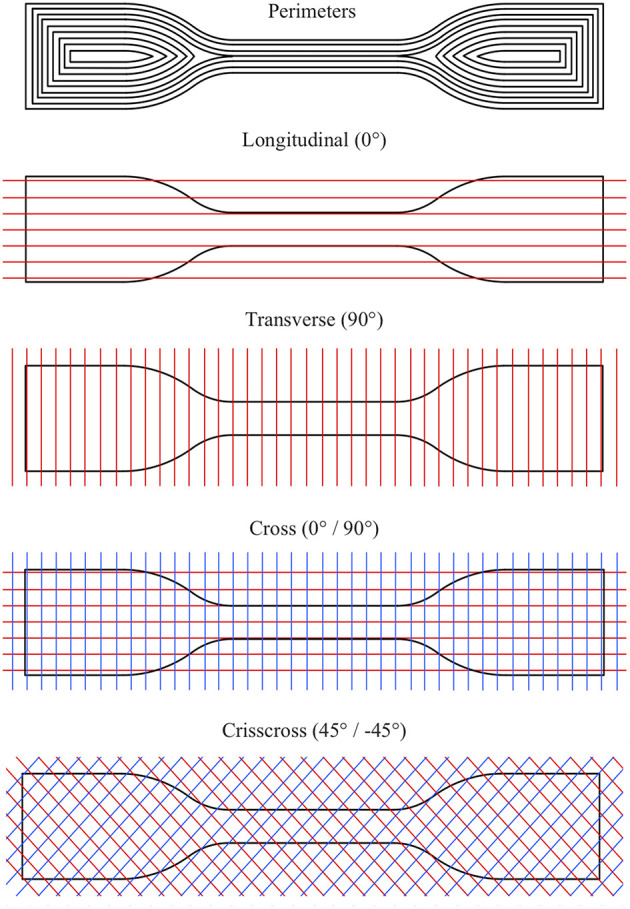
The print direction of the dumbbell test pieces fabricated from PDMS.

To further investigate performance differences, we analyzed the stiffness change of the PDMS material caused by used fabrication methods. Sixty six dumbbell test samples were prepared and divided into 11 subgroups in order to measure the effects of fabrication methods, nozzle size and print direction on the Young's modulus of the used PDMS material. Twenty four of the samples were molded and 42 of them were 3D printed by following the ASTM D412 type C dimensions. Molded test samples were divided into 4 subgroups depending on their cure time: DS10-slow, DS10-medium, DS10-fast, and DS10-very-fast. 3D printed samples were fabricated only with DS10-very-fast and were divided into 7 subgroups depending on their print directions and nozzle sizes: perimeters, longitudinal, transverse, cross, crisscross with 0.417, 0.839, and 1.019 mm (Figure 6). Per each subgroup 6 samples were fabricated and defective ones were eliminated; at maximum 3 samples were eliminated from each subgroup. For all the test samples, the main composition (DS10-very-fast with 1 wt % Thi-Vex additive) was mixed with 10 wt % thinner (Walker et al., 2018) and none of the samples were degassed. Print parameters for the dumbbell test samples can be seen in Supplementary Table 3.

After waiting overnight to ensure samples reached their final mechanical properties, prepared samples were attached to a Mark-10 motorized test stand including 1000N load cell (MR01-200-1, Mark-10, NY) for measurements (Figure 4A). Dumbbell pieces were pulled up with a speed of 60 mm/min until failure. Due to sensor resolution, a systematic error of ±0.5N was introduced into all test results. The Young's modulus was calculated by fitting a 100% tensile modulus line into the experimental data (Supplementary Figure 6).

## Results and Discussion

In this section, first, we present the changes in the mechanical properties of PDMS samples after gamma irradiation and analyze the potential of the soft robots under radiation environments. Then, we verify the viability of soft robots under 3 selected radiation environments based on potential tasks in the nuclear industry. Later, we present the absorbed gamma irradiation by the 3D printed hexapus robot and its estimate operation time. We fabricated the hexapus robot by taking advantage of AM to avoid many fabrication challenges inherent to the molding techniques due to the complex design of the robot. To verify the 3D printed robot's reliability, we present their performance differences with molded counterparts. Finally, to explain performance differences, we show the effects of fabrication methods, nozzle sizes, and print directions on the stiffness of the fabricated PDMS material.

### Changes in the Mechanical Properties of PDMS After Gamma Irradiation

**Figure 7:**
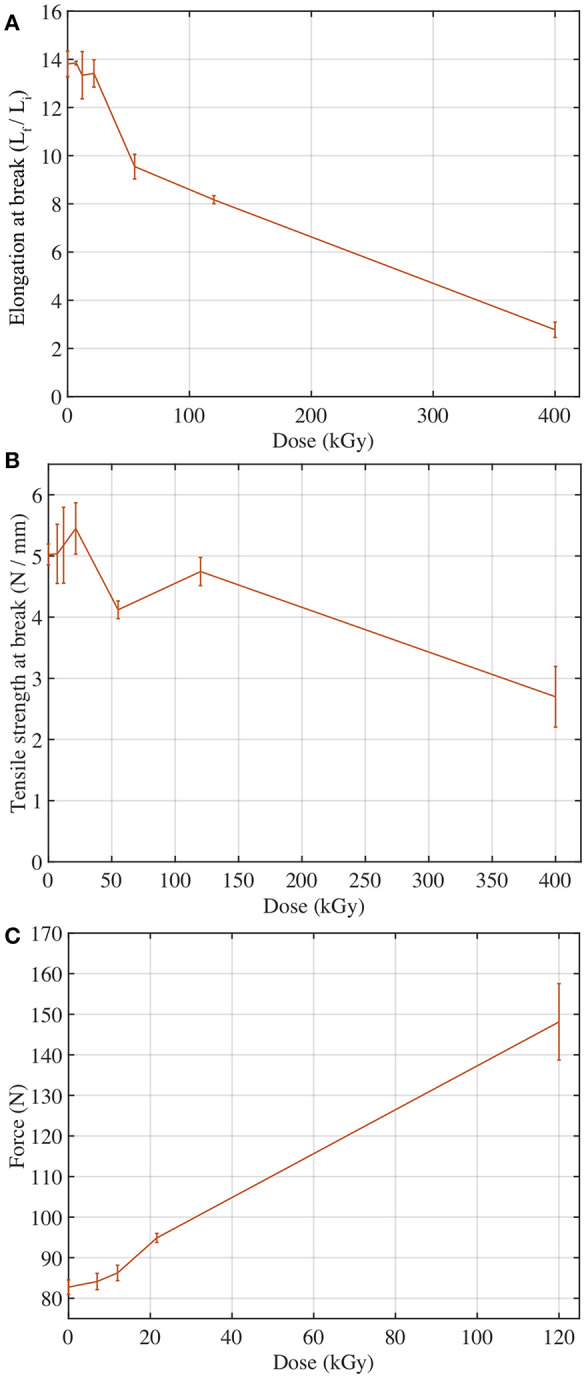
Changes in the mechanical properties of PDMS after gamma irradiation (Oshiro, 2018). **(A)** PDMS elongation decreases with increasing gamma dose. **(B)** PDMS tensile strength for increasing gamma dose. **(C)** Force at 25% compressive strain with increasing gamma dose.

The measured changes in elongation at break, tensile strength, and compression were plotted as a function of cumulative dose in Figure 7. Results indicate that increased cumulative gamma dose leads to decreased elongation at break (Figure 7A). However, the relationship is not strictly linear. From 7 to 21.6 kGy, elongation at break decreases slowly, remaining nearly constant. Above 21.6 kGy, there is a steep decrease in elongation at break up to the highest measured dose, 400 kGy. This agrees with past literature, which shows either a small initial increase (Warrick, 1955) or slight decrease (Van de Voorde and Restat, 1972; McCarthy and Mark, 1998) followed by an eventual decrease in elongation at break at higher doses. For the tensile strength property of the material, results showed a slight initial increase from 0 to 21.6 kGy followed by an overall decrease in tensile strength (Figure 7B). This includes a steep drop in tensile strength at 55 kGy followed by a recovery back to the general decrease trend from 120 to 400 kGy. The overall trend, not including the 55 kGy drop, agrees with results from a CERN technical report. Past results do not agree on the overall effects of gamma radiation on tensile strength; Warrick (Warrick, 1955) showed an initial increase followed by a sharp decrease while McCarthy and Mark (1998) showed a constant tensile strength over the range of 200–400 kGy. This may be explained by the difference in the experimental aims of the past two studies and this current one. Where Warrick and McCarthy sought an optimum dose to vulcanize the rubber, this study focuses on already cured, solid silicone rubbers. This suggests that gamma irradiation improves the tensile strength of the uncured or incompletely cured material until it achieves a maximum, after which the molecular-level effects become detrimental rather than curative. Thus, the silicone rubber studied here improves to its maximum at roughly 12 kGy then degrades as dose increases above 20 kGy. We anticipate that these results would apply for common cured PDMS materials. However, further testing is required to verify. The results from the CERN technical report fit this profile and support this conclusion (Voorde and Restat, 1972). Since the overall effects of gamma radiation on PDMS in this experiment are decreased elongation at break and decreased tensile strength, it indicates molecular crosslinking is likely the dominant effect within the PDMS matrix as doses are increased. While this experiment did not include investigation on a molecular level and therefore cannot confirm this overall trend with certainty, it does agree with past research by Hill which suggests that irradiation of PDMS results in a higher crosslinking yield than scission yield (Hill et al., 2000). However, an explanation of molecular effects is not necessary to extend the mechanical results and their influence on potential usage areas.

The compression tests of the cylindrical samples determined stiffness by measuring the force required to compress the disc to 75% of its original height, shown in Figure 7C. These results point to an increase in stiffness as cumulative dose increases. This increase in stiffness is likely due to a direct response relationship between radiation exposure and cross-linking. This agrees with Basfar's research that shows beta radiation at similar doses results in increased crosslinking and increased resistance to compression (Basfar, 1997). While Basfar's experiment sought to determine the dose required to completely cure a liquid silicone rubber to solid state using radiation, it still indicates that the dominant effect of cumulative radiation is increased crosslinking, which is consistent with the current evaluation of PDMS. Regarding any thickness dependent variations in property degradation of both dumbbell and compression samples, based on the 1.25 MeV photon energy of the Co-60 irradiator, it is safe to assume the dose is uniformly distributed. 10 cm of PDMS reduces the absorbed dose by ~25%. 3.8 cm is required to alter the absorbed dose rate by 10%, which indicates our hexapus design is also safe. Higher energy photons would require larger thicknesses, up to 18 cm for 10 MeV photons.

The effect of temperature on PDMS has been previously evaluated in the literature (Camino et al., 2001, 2002) and is known to have an influence and was not considered in this study as all irradiations were performed at room temperature. Based on the differences in damage mechanisms between thermal and radiation exposure, it is not anticipated that these effects would interact; however, this has not been evaluated.

Overall, with increasing exposure to high dose gamma radiation, the mechanical properties of PDMS decrease in functionality, as expected. The results of the elongation at break tests suggest that material performance is not greatly impacted up to 20 kGy, at which point it begins to lose its ability to extend more drastically. Similarly, the results of the tensile strength tests suggest that performance is minimally impacted up to roughly 20 kGy followed by a gradual decrease at higher doses. The stiffness of the material increases steadily as the cumulative dose increases. These results are consistent with the theory (Hill et al., 2000) that both scission and crosslinking occur initially while crosslinking becomes the dominant effect of gamma radiation on PDMS beyond 20–50 kGy. The major concerns for soft robots and their manipulators at these higher doses is that more pressure will likely to be needed to maintain the range of motion.

### Viability of Soft Robots Under Selected Radiation Environments

In order to translate the functionality of soft robotics for potential tasks in the nuclear industry, the cumulative dose at each radiation environment was evaluated for its resultant change to the material properties of PDMS. This study provides a preliminary evaluation of the properties of PDMS under certain irradiation conditions. A fully functional test of the robot under inflation or with a load was not possible in the available irradiation facilities. Additionally, any electronics required for actuation of the robot would not survive these doses (up to 400 kGy) and were therefore not used. The main reason for testing a very high dose rate (400 kGy) is to represent very long time effect on property degradation.

**Figure 8:**
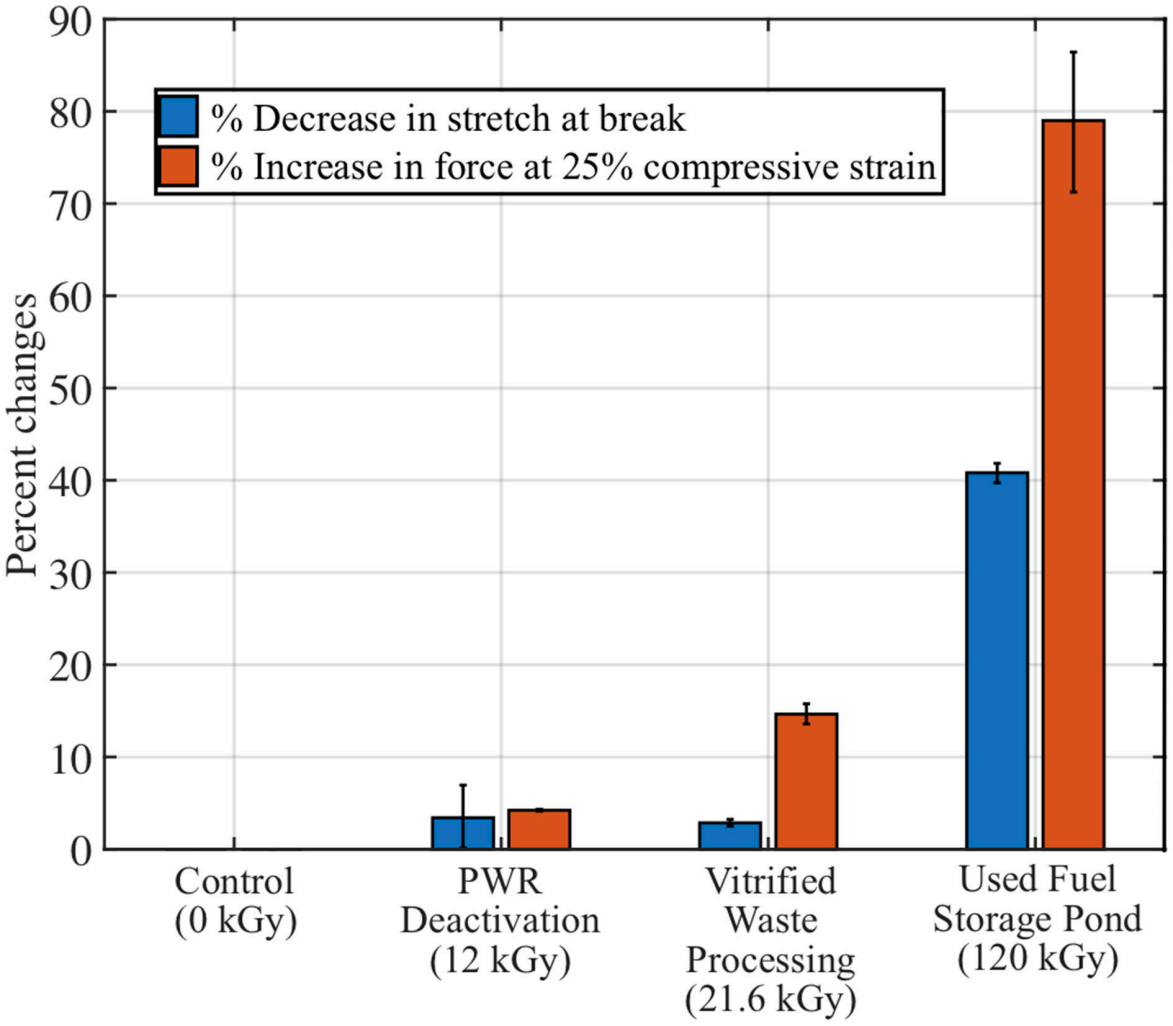
Fractional changes to PDMS mechanical properties compared to representative gamma environments (Oshiro, 2018).

A complex soft robot geometry with external loads could have a state of strain that is a combination of uniaxial, biaxial, shear, and volumetric strains, and full failure characterization requires a suite of tests. However, due to the limitations of this study, mentioned above, detailed failure characterization including equibiaxial strain tests at the inflation state could not be performed. Instead, elongation at break and stiffness were used as measures of mechanical changes due to their predictable effects and direct relation to the functionality of the material. As a function of dose, the fractional change to each property was measured by taking the difference between the irradiated and control sample values and dividing by the control sample value. As shown in Figure 8, compression changed by more than 50% in the used fuel pool, and elongation at break in the used fuel pool changed by more than 25%. A similar polymer radiation study considered 50% change as a benchmark to assess the viability of a material (Bonin et al., 2004). By this rubric, soft robotic systems made out of PDMS materials are viable in most radiation environments, which is promising. Changes to the mechanical properties will result in some corresponding loss of function but understanding these mechanical changes as a function of exposure may allow for control systems to compensate for reliable and consistent performance of the soft robots.

### Operation Time Estimation and Absorbed Gamma Dose Rate of 3D Printed Hexapus Robot Under Radiation

**Figure 9:**
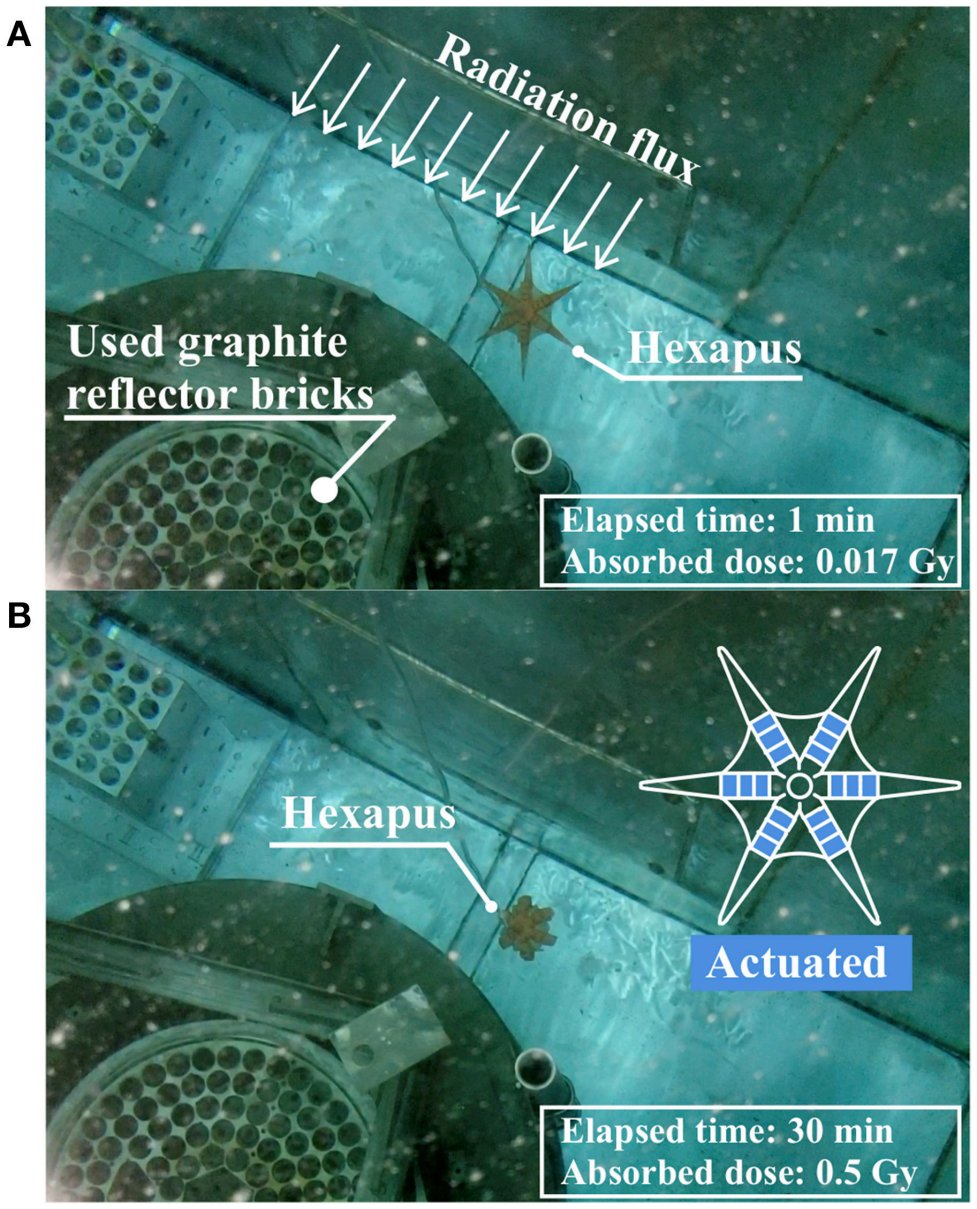
Actuation of the hexapus robot while absorbing gamma irradiation inside bulk shield tank for 30 min. **(A)** Hexapus nominal state. **(B)** Hexapus actuated state.

After submerging the 3D printed hexapus robot to estimate the operation time, the underwater ion chamber was also submerged directly next to it and measured the gamma dose rate as 1 Gy/h. This measured dose rate does not account for the neutron flux coming from the neighboring reactor, which cannot be directly measured under water. A conservative estimate assumes the 1 Gy/h dose rate in which the robot was exposed for 30 min while cycling through its push and pull motions, receiving a total dose of 0.5 Gy. A benchmark of 50% change in material properties is used in polymer radiation experiments to assess the viability of the material (Bonin et al., 2004). While the stiffness changes around 50% at 70 kGy, elongation does not reach the 50% change metric until 120 kGy and the tensile strength is even less susceptible to change. Therefore, it is speculated that the hexapus robot could operate around 70,000 h in the radiation environment shown in Figure 9 before its stiffness changed more than 50%, assuming with a dose rate of 1 Gy/h. Also considering the fractional changes to the PDMS mechanical properties in Figure 8, the robot could operate for the 12-h task time in two of the three proposed radiation environments. The highest dose rate environment, the used fuel storage pond, would have a reduced task time on the order of 7 h in which the stiffness changes by ~50%. These estimates assume the rest of the robot's control system and pumps continue to perform.

### Robot Performance Comparison: 3D Printed vs. Molded

**Figure 10:**
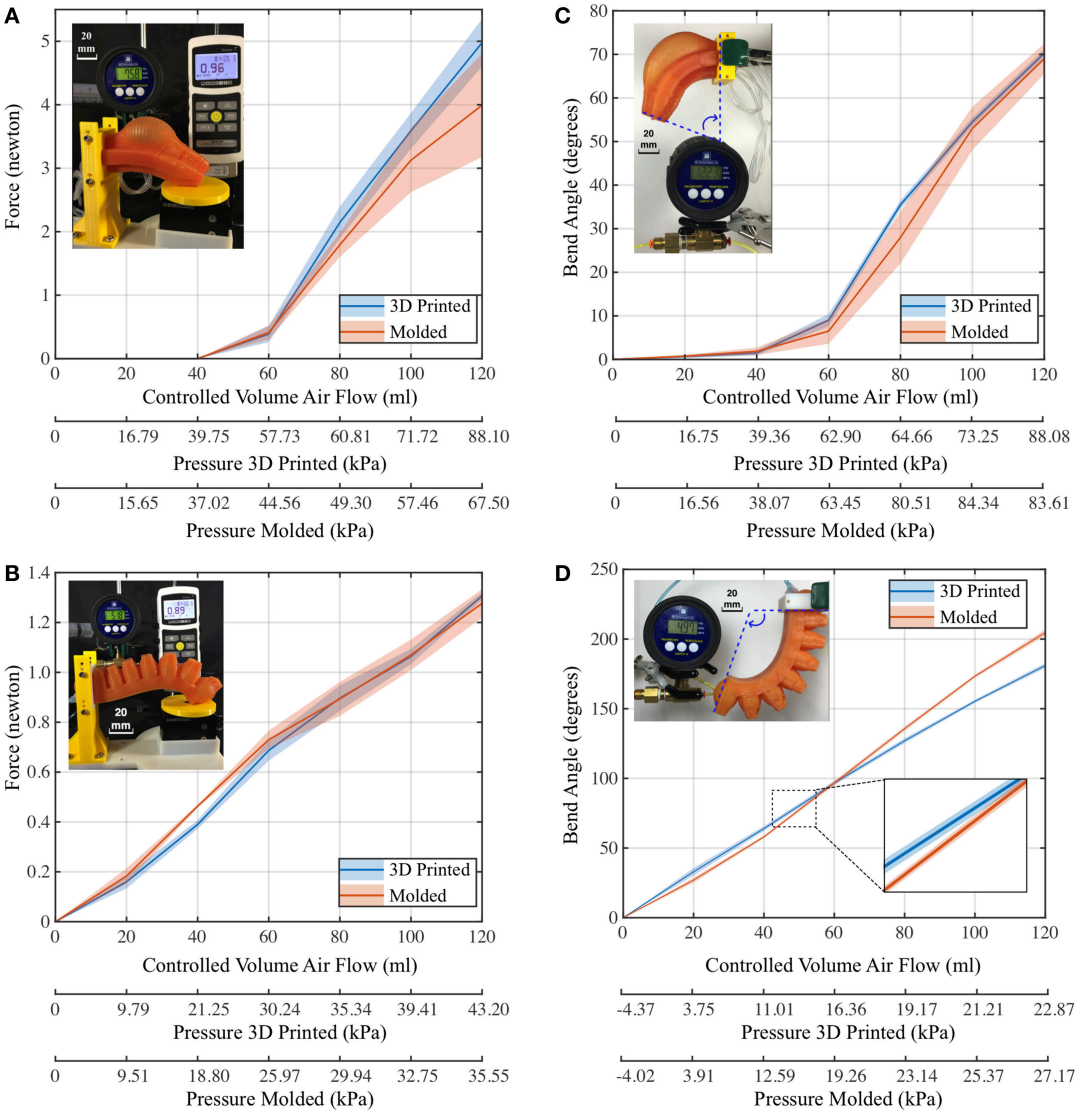
Performance comparison results (Yirmibesoglu et al., 2018). **(A)** Blocked force comparison (4 channel tentacles). **(B)** Blocked force comparison (Pneu-net actuators). **(C)** Bend angle comparison (4 channel tentacles). **(D)** Bend angle comparison (Pneu-net actuators).

The results of the blocked force and bend angle tests are shown in Figure 10. Plotted values reflect the average of the experimental data while the shadowed regions represent one standard deviation. For the 4 channel actuator, we collected data from each channel (*n* = 4), and for Pneu-net actuator, we collected data from 3 trials (*n* = 3). The results of the blocked force tests for the 4 channel tentacles (Figure 10A) showed that the force applied by the molded version varies more with increased pressure. Moreover, when the tentacle attachment height above the sensor surface increased, for a fixed inflation volume, the measured maximum blocked force increased from 1 to 2 N (Supplementary Figure 7). At a 15 mm height, the 3D printed 4 channel tentacle was able to apply more force than its molded counterpart. When we investigated accumulated pressure levels inside the channels, an injection of 120 ml of air resulted in a pressure of 88 kPa for 3D printed tentacle, but only a 67.5 kPa pressure for the molded tentacle. The results of the blocked force tests for Pneu-net actuators (Figure 10B) showed that both the 3D printed and molded actuators applied the same amount of force (with a 0.03 N difference in between). However, the blocked force values of the molded version again varied more with increased pressure. Also, when we analyzed the data regarding the pressure levels, the molded actuator applied the same amount of force but with 10 kPa less accumulated pressure. The molded actuator did incur a failure at the seam position and was repaired to continue with the experiments.

The results of the bend angle tests for 4 channel tentacles (Figure 10C) showed that both tentacles bend to around the same angle. However, it can be seen that the internal channel pressure of the molded actuator jump by 20 kPa when 80 ml of air is injected. The results of the bend angle tests for Pneu-net actuators (Figure 10D) showed that after 60 ml of airflow, the angle difference between actuators increased to 23.7°, with molded actuator achieving a higher bend angle. However, this time the bend angle results of both actuators varied less in between experiments.

**Figure 11:**
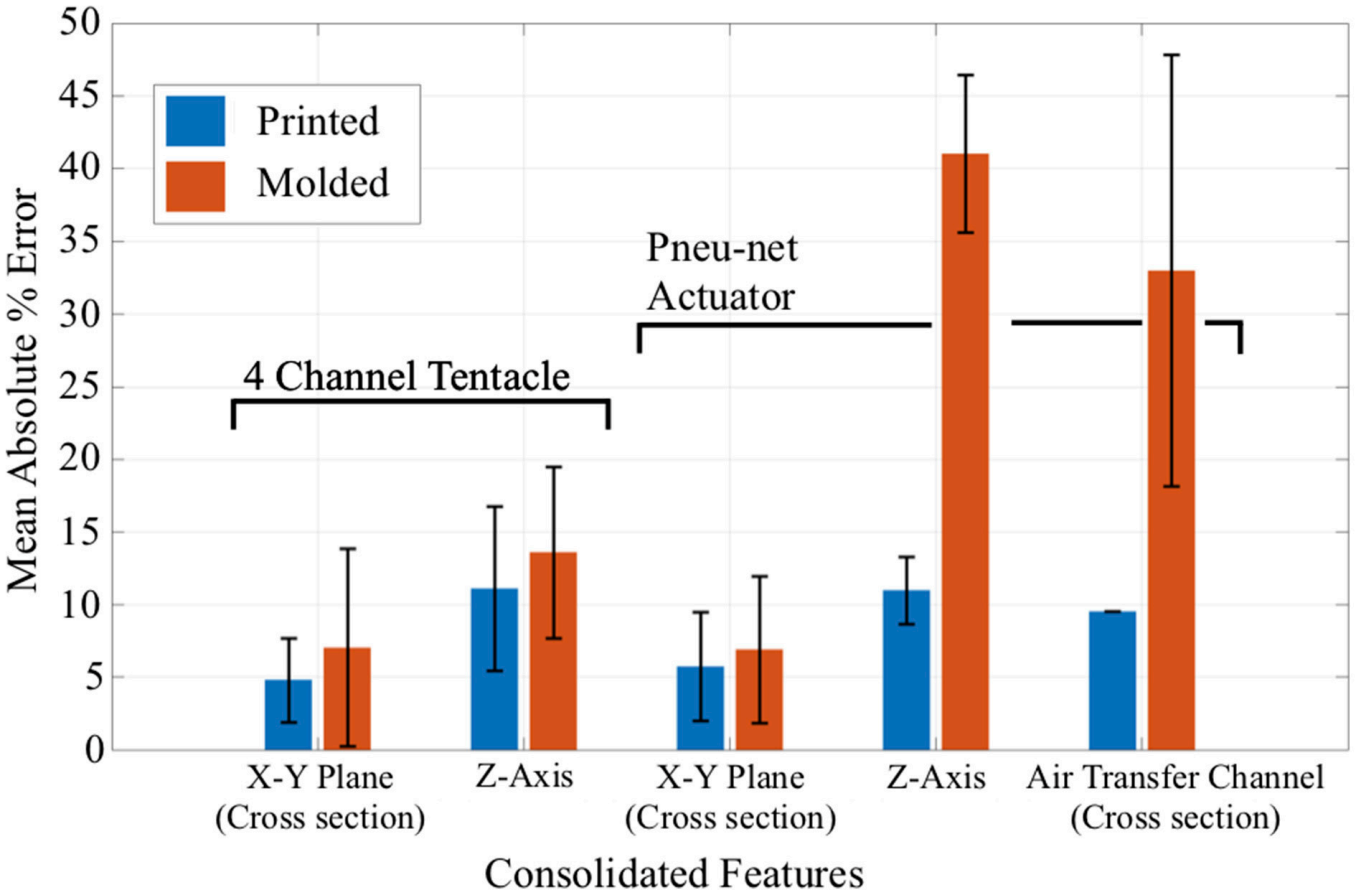
Dimensional quality comparison (Yirmibesoglu et al., 2018).

To explain and discuss the performance differences between the 3D printed and molded soft robots, first, we conducted dimensional error analyses. Results are shown in Figure 11 with the consolidated feature groups for both the 4 channel tentacles and the Pneu-net actuators in terms of percent error deviations. The 3D printed, 4 channel tentacle and the Pneu-net actuator deviated on average less from the original dimensions provided from the CAD file compared to their molded counterparts. The 3D printed tentacle has a smaller standard deviation than the molded one for cross-sectional features, while the standard deviations for vertical features for both robots are about the same. The difference in applied force across the two fabrication methods could potentially be caused by a change in stiffness resulting from each fabrication method. Therefore, we analyzed the effects of the fabrication method on the stiffness of the PDMS material, reported in the next section. The observed difference in the accumulated pressure levels inside the channels is likely the result of the inward warping of the wax cores used to create the long channels in the molded tentacle (Supplementary Figure 8). Also, since we do not have a force plate blocking the motion in the bend angle tests, compared to the blocked force tests, we believe that an uneven geometry of the channel cross-sections may have caused the 20 kPa jump (Supplementary Figure 8).

Regarding the Pneu-net actuators, both the 3D printed and molded actuator's cross-sectional errors were comparable. However, for both the z-axis error and the air transfer channel errors, the molded Pneu-net performed poorly because these dimensions were significantly affected by the manual process of adhering the top and bottom layers of the actuator (lamination casting). This manual process caused uneven geometry at the lamination layer. The bottom section of the molded Pneu-net actuator was thinner compared to the 3D printed actuator. As a result, we observed a 23.7° bend angle difference between the two actuators.

Overall, performance results of the 3D printed 4 channel tentacle and Pneu-net actuator varied less compared to their molded counterparts. The dimensional error results in the cross-sectional areas, which directly affect the performance, were similar for both the molded and 3D printed robots. Moreover, the wall thicknesses of the molded actuators were thicker than the 3D printed ones because the elastomer expands with the mold during the curing process. The dimensional comparison results suggested that the 3D printed soft robots should expand more, as they had thinner walls compared to the molded counterparts, but the experiments showed the opposite. Due to these observations, the following section describes a stiffness analysis that was performed to resolve this discrepancy.

### Effects of Fabrication Methods, Nozzle Size, and Print Direction on the Stiffness of PDMS Material

In this section, we first focused on the effects of fabrication methods (molding vs. 3D printing) on the stiffness of PDMS material which is directly related to the performance differences observed in the previous section. We then investigated the effects of nozzle size and print direction (Figure 6) on the stiffness of PDMS material to understand if the stiffness property of the PDMS can be changed by these specific parameters.

In Table 1 we compared the effects of fabrication methods with different available cure times^2^ on the Young's modulus of the same material (DS10). For the molding method, we compared the effects of slow, medium, fast and very fast cure times on the stiffness of DS10. For 3D printing method, we only used very fast cure time and compared the stiffness result with its molded counterpart. The results for the molding method with different cure times (Table 1) show that DS10-slow has the highest Young's modulus. However, the wait time for the material to settle to its final mechanical properties is 7 h. In contrast to the molding method, the use of 3D printing method to fabricate the same object with DS10-very-fast provides equivalent or better stiffness results with faster fabrication times.

**Table 1:** Effects of fabrication method on Young's modulus with different available cure times.

**Dragon Skin 10 product line and fabrication method**	**Cure time^2^ (h)**	**Young's modulus (kPa)**	**Standard deviation (kPa)**
Slow (molded)	7	135.9	4.3
Medium (molded)	5	108.3	5.4
Fast (molded)	1.25	100.0	12.3
Very fast (molded)	0.5	128.9	10.1
Very fast (3D printed–crisscross−0.839 mm nozzle)	0.5	147.0	11.5

Even though we used the same materials for the different fabrication techniques of the 4 channel tentacles and Pneu-net actuators, there was only one difference that we neglected in our previous work (Yirmibesoglu et al., 2018). Since DS10-very-fast material's pot-life was 4 min, it was not possible to mold the actuator designs. Despite our molding experience, the material would begin to cure before fully filling the mold and settling. Due to this fact, and the indication from the manufacturer that DS10 product lines have the same mechanical properties, we used DS10-slow for fabricating the molded counterparts. With the results on cure time from Table 1, we verified that the use of DS10-slow or very-fast materials with the molding method does not necessarily change the overall actuator stiffness. The Young's modulus measurements for DS10-slow and DS10-very-fast are within a standard deviation of each other. However, the use of DS10-very-fast with the 3D printing method might have increased the stiffness of the fabricated soft robots due to a small region of overlap (135.5–140.2 kPa) between the Young's modulus measurements. The maximum Young's modulus of the molded DS10-slow material was 140.2 kPa whereas the maximum Young's modulus for the 3D printed DS10-very-fast material was 158.5 kPa. The 3D printed actuators had a higher stiffness, resulting in the observed performance differences reported in the previous section. The molded 4 channel tentacle could not apply the same force as the 3D printed counterpart because it was less stiff, and in the case of Pneu-net actuator, since the molded actuator is less stiff than the 3D printed one, it achieved higher bend angle.

The results illustrated in Table 2 clearly indicate that when the nozzle size decreases the printed material becomes stiffer. The increase in stiffness could be due to preferential polymer chain alignments caused by the decrease in nozzle diameter, but these effects were not studied as they were not within the scope of the work. The amount of micro air bubbles introduced into the material and the total weight of each printed test samples may play a role in the final results as well. We recorded the weights of each dumbbell test samples to make sure that there was not an outlier in each category shown in Supplementary Table 6. At the moment, we cannot compare the significance of these 3 variables: polymer chain alignment, air bubbles, and the total weight of the samples on the final results seen in Table 2.

**Table 2:** Effects of nozzle size on Young's modulus.

**Nozzle size (mm)**	**Young's modulus (kPa)**	**Standard deviation (kPa)**
0.417	160.8	6.5
0.839	147.0	11.5
1.019	96.5	5.1

Young's modulus results for different print directions with a fixed nozzle size are shown in Table 3. The standard deviation ranges for each category overlap the measured values; thus, we cannot distinguish the effects of different print directions on the stiffness of the test pieces. Unlike the thermoplastic polyurethane (TPU) material which causes 3D printed actuators to have anisotropic properties (Fernandez-Vicente et al., 2016; Yap et al., 2016), thermoset PDMS material seems to maintain its isotropy in the 3D printing process. Therefore, contrary to TPU material, print direction does not significantly affect the stiffness of the final PDMS product at to 0.839 mm nozzle size. It can be assumed that down to 0.839 mm nozzle size, use of different print directions will not affect the stiffness of the final PDMS product. However, when the nozzle size further decreases the preferential alignment of the polymer chains of the PDMS material may cause print directions to change stiffness. This result also provides an opportunity to choose an appropriate print direction to increase the surface finish quality of the fabricated parts without compromising the stiffness (Supplementary Figure 9).

**Table 3:** Effects of print direction on Young's modulus.

**Print directions (at 0.839 mm nozzle size)**	**Young's modulus (kPa)**	**Standard deviation (kPa)**
Perimeters	147.7	4.1
Longitudinal	140.5	11.0
Transverse	152.1	15.6
Cross (0°/90°)	145.3	4.8
Crisscross (45°/−45°)	147.0	11.5

In summary, sections Robot performance comparison: 3D printed vs. molded and Effects of fabrication methods, nozzle size, and print direction on the stiffness of PDMS material support that the 3D printing method with the use of small nozzle sizes increased the stiffness of the fabricated soft robots and maintained more accurate dimensions as defined in the CAD models. These results lead 3D printed soft robots to perform better than or equal to their molded counterparts while being more reliable and robust. Verifying the reliable operation of the 3D printed soft robots will allow more soft robotic applications to emerge under radiation environments.

## Conclusion

In order to assess the usefulness of soft robots in radiation environments, changes in the mechanical properties such as elongation, tensile strength, and compression of the PDMS material were measured after gamma irradiation. We analyzed the viability of soft robots under 3 radiation environments selected from the nuclear power industry. Finally, we submerged and operated a 3D printed soft robot in a radiation environment and measured the absorbed dose rate to estimate its operation time. To ensure the reliability of the 3D printed soft robots and verify their performances, we compared them with their molded counterparts; the blocked force and the bend angle experiments were tested on four channel tentacle and Pneu-net actuators. To analyze performance results in detail, we also investigated dimensional errors and the effects of fabrication methods, nozzle size, and print direction on the stiffness of PDMS material.

The preliminary evaluation of the properties of PDMS under certain irradiation conditions concludes that with increasing exposure to gamma irradiation, the mechanical properties of PDMS decreased in functionality. However, up to 20 kGy gamma radiation, the elongation and tensile strength of the material are minimally impacted. Considering the fractional changes to the PDMS mechanical properties, it is safe to assume that soft robots could operate for the 12-h task time in two of the three proposed radiation environments. Also, the 3D printing method increased the stiffness of the fabricated soft robots and maintained more accurate dimensions as defined in the CAD models. Therefore, 3D printed soft robots performed better than or equal to their molded counterparts while being more reliable and robust.

The main limitation of this study was due to the difficulties of experimenting under gamma irradiation. We were unable to quantify the performance change of the hexapus robot due to lack of camera equipment that can work in underwater radiation environments. Additionally, any electronics required for actuation of the robot would not survive doses up to 400 kGy; thus detailed failure characterization including equibiaxial strain tests at the inflation state could not be performed. Instead, elongation at break and stiffness were used as measures of mechanical changes due to their predictable effects and direct relation to the functionality of the PDMS material. Even with these limitations, this study provides a preliminary method for assessing the potential of soft robots in radiation environments. While full functional tests will be required to deploy soft robots in nuclear environments, current findings show great promise for soft robots in high dose radiation environments. Future work will focus on quantifying the functionality of a 3D printed soft robot outside of an aquatic environment under radiation with radiation hardened test equipment.

The authors believe that soft robots under radiation environments warrant further study since the mechanical properties of the PDMS material studied showed great promise under radiation. Advantages provided by 3D printing of PDMS will give the opportunity to design and fabricate more complex robots for the soft robotics community. Future developments in this field will allow researchers to broaden the application fields of soft robotics.

## Author Contributions

OY is the main author who designed and developed the 3D silicone printer and carried on the experiments, sample preparations, 3D printing soft robots, and their analysis. He prepared the draft as the main author. Also, he conducted the irradiation experiment for the hexapus robot. TO is the second author who prepared radiation test samples, conducted irradiation tests and interpreted results for the radiation environments. Sections from his thesis are used as subsections of the manuscript. GO is the third author who assisted with experiment design and interpretation of the results. CP supervised radiation research studies and provided helpful comments. YM supervised the research, guided its progress, provided helpful comments as main author's Ph.D. supervisor.

### Conflict of Interest Statement

The authors declare that the research was conducted in the absence of any commercial or financial relationships that could be construed as a potential conflict of interest.

## Footnotes

^1^https://softroboticstoolkit.com/3d-silicone-printer (accessed April 2019)

^2^https://www.smooth-on.com/product-line/dragon-skin/ (accessed: April 2019)
