# Supplementary material for: Evaluation of 3D Printed Soft Robots in Radiation Environments and Comparison With Molded Counterparts
Source: Front Robot AI. 2019 May 24;6:40. doi: 10.3389/frobt.2019.00040 (PMC7805716; doi:10.3389/frobt.2019.00040)
Supplement: Supplementary file 2 [file Data_Sheet_1.PDF]

frontiers

Supplementary Material

3D Printed Soft Robots in Radiation Environments

Osman Dogan Yirmibesoglu*, Tyler Oshiro, Gina Olson, Camille Palmer, Yigit Menguc

*** Correspondence:** Corresponding Author: yirmibeo@oregonstate.edu

1 Table of acronyms used in the paper

Supplementary Table 1. Table of acronyms in alphabetical order.

Acronyms	Definitions	Acronyms	Definitions
AM	Additive Manufacturing	PDMS	Polydimethylsiloxane
CAD	Computer-Aided Drawing	PWR	Pressurized Water Reactor
CoRIS	Collaborative Robotics and Intelligent Systems	RFT	Robotics Fast Track
DIW	Direct Ink Writing	SLA	Stereolithography
DOF	Degrees of Freedom	TMI-2	Three Mile Island, reactor 2
DS10	Dragon Skin 10	TPU	Thermoplastic polyurethane
FFF	Fused Filament Fabrication		

2 3D printer setup

We designed a custom 3D printer with a broader workspace area (40 x 40 x 100 cm - width x height x depth) compared to available open source FDM printers on the market. The 3D printer design can be seen in **Figure 2 A**.

2.1 Printer gantry and control

The supporting frame of the printer was built with T-slotted aluminum profiles and corner brackets (80/20 Inc, IN). Gantry operation was accomplished with common bipolar stepper motors (Stepper online, China), with model NEMA 17 for x- and y-axis operation and model NEMA 23 for z-axis operation. A Smoothieboard 5C (Uberclock, OR) served as the printer motherboard.

2.2 Software

We used 'Pronterface' as the printer controller program, with a second software called 'Slic3r' to generate motor control commands (G-code) from STL file formats and determine the print sequence. The printing process could be started by uploading the G-code file exported from Slic3r into Pronterface.

2.3 Syringe pump

Two high precision syringe pumps are used (PHD ULTRA, Harvard Apparatus, MA) in combination with our custom 3D printer. The syringe pump feeds the silicone materials (silicone base and silicone crosslinker) at a constant rate into the extruder mechanism. Tubing used for material flow was coupled with tube-to-tube wye connectors (3/16", McMaster-Carr, CA) with 45° separation for easy cartridge replacement during longer print times.

2.4 Extruder mechanism

The final extruder consisted of a six-part modular design for easy cleaning, shown in **Figure 3 B**. Equal amounts of silicone base and crosslinker flow from separate lines into the mixer. As the two parts of silicone come into the top section, they are mixed by the reamer and forced downward by the syringe pump pressure. The mixer assembly, similar to the design used for active mixing of complex fluids at micro-scale (Ober et al., 2015), is powered by a 12V DC motor (Micro Metal Gearmotor, Pololu, NV). Nozzles with inner diameters of 0.417 mm, 0.839 mm, and 1.019 mm (MT25-PBN, Techcon, CA) were used to achieve different print resolutions. Extruder parts were manufactured with a high precision 3D printer (Form2, Formlabs, MA). The step-by-step guide to manufacturing the initial version of this extruder mechanism is publicly available on the Soft Robotics Toolkit[1].

---

[1] https://softroboticstoolkit.com/3d-silicone-printer (Accessed: April 2019)

2.5 Benchmark study

Supplementary Table 2. Preliminary comparison between the specifications of common 3D printers that can fabricate soft materials.

3D printers for fabricating soft materials	Built volume	Layer height (for the used soft material)	The softness of the print material	Support generation	Print technology
Authors' 3D Silicone Printer	400 x 400 x 1000 mm	400 microns	10A Shore durometer (Dragon Skin)	No	Direct ink writing
Picsima[2]	unavailable	400 microns	10A Shore durometer	Yes	Sub-surface catalyzation
Stratasys Objet500 connex1[3]	490 x 390 x 200 mm	16 microns	27A Shore durometer (TangoBlack)	Yes	PolyJet
Carbon 3D M2[4]	189 x 118 x 326 mm	75 microns	35A Shore durometer	Yes	Carbon's proprietary CLIP process
Formlabs form2[5]	145 × 145 × 175 mm	100 microns	50A Shore durometer	Yes	SLA
Ultimaker S5[6]	330 x 240 x 300 mm	200 - 20 micron	85 A Shoe durometer (Ninjaflex[7])	Yes	Fused filament fabrication

[2] Picsima: https://3dprintingindustry.com/news/picsima-3d-print-silicones-without-support-structures-34672/ (Accessed: January 2019)

[3] Stratasys: https://www.stratasys.com/polyjet-systems (Accessed: January 2019)

[4] Carbon 3D: https://www.carbon3d.com/ (Accessed: January 2019)

[5] Formlabs: https://formlabs.com/3d-printers/form-2/ (Accessed: January 2019)

[6] Ultimaker: https://ultimaker.com/en/products/ultimaker-s5/specifications (Accessed: January 2019)

[7] NinjaTek: https://ninjatek.com/ (Accessed: January 2019)

3 Effects of adding thinner to the print formula and change in tube length on the accumulated load of the syringe pumps

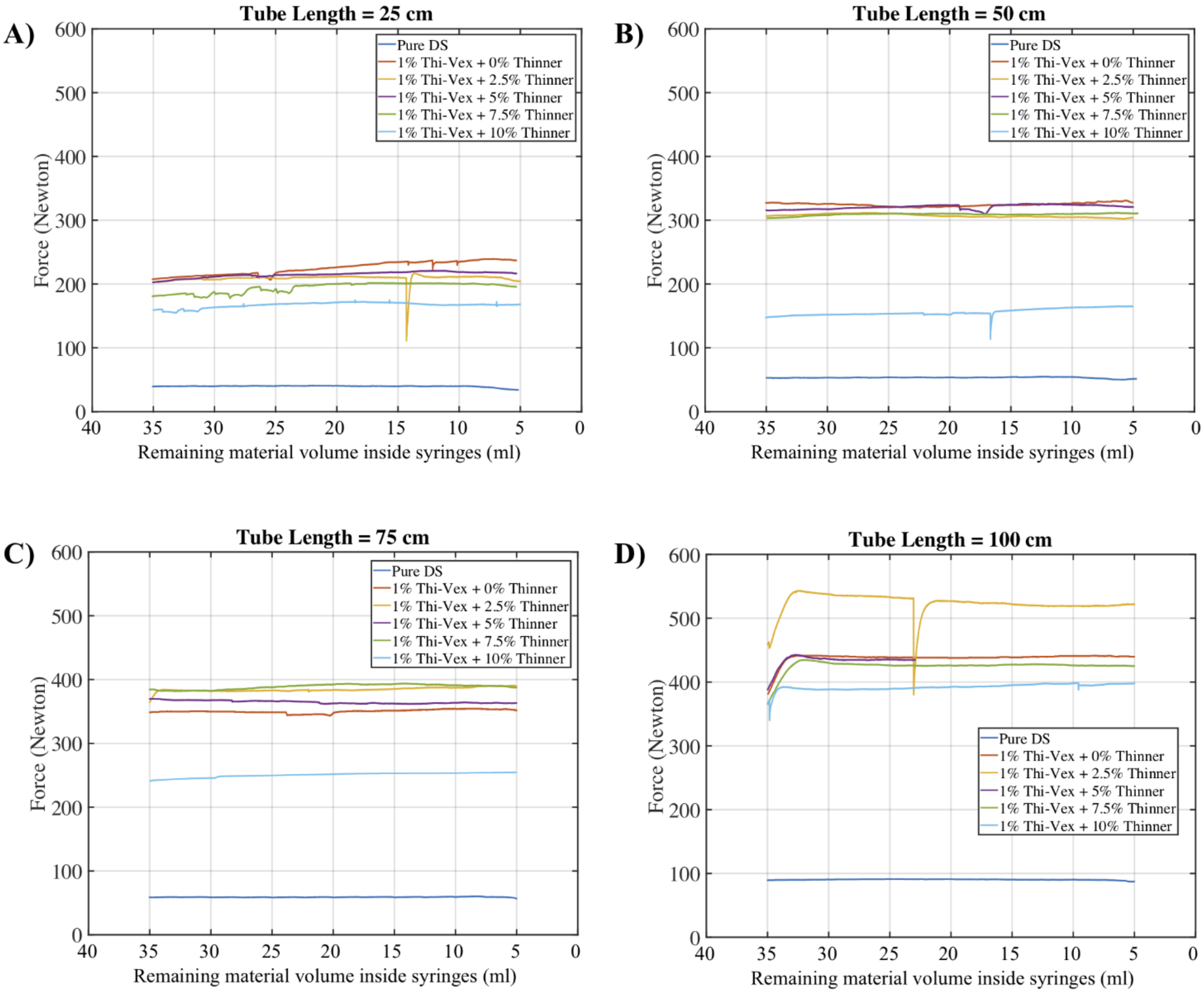

Supplementary Figure 1. Accumulated load on syringe pumps based on changes of tube length and print formula. **A)** Tube length = 25 cm, **B)** Tube length = 50 cm, **C)** Tube length = 75 cm, **D)** Tube length = 100 cm.

For each experiment, two 60 ml syringes filled with print material were attached to the experimental setup shown in **Supplementary Figure 2**. In total, of 24 syringe pairs were tested with 6 different material compositions at 4 different tube lengths. Results are shown in **Supplementary Figure 1**. As a control sample, we plotted pure DS10 material in all graphs. By just looking into DS10 pure (no addition of Thi-Vex or Thinner) we can observe the effects of tube length isolated from the effects of Thi-Vex and Thinner. As tube length increased from 25 cm to 100 cm, it caused an increase in the force required to maintain the flow of the silicone material from around 40.4 N to 91 N **Supplementary Figure 1 A and D**. When we only added Thi-Vex material to increase print fidelity, at the same tube length (**Supplementary Figure 1 A)**, the required force increased from around 40.4 N to 230 N, almost 6 times higher. When the effects of Thi-Vex were combined with the increasing tube length, the required force increased to 438 N **Supplementary Figure 1 D**. Addition of material thinner was

required to reduce the amount of load accumulated on the syringe pumps and determining the trend of shorter tubing reducing load was significant. Due to the large print volume we have **(Figure 2 A),** the minimum tube length we could use was around 75 cm. Addition of 10 weight % thinner material lowered the required force values from around 351 N down to 252 N at the selected 75 cm tube length (**Supplementary Figure 1 C)**. Anything less than the 10 weight percent thinner added showed no significant decrease in force values. In **Supplementary Figure 1 A, B, C, and D** line plots show 0%, 2.5%, 5%, 7.5% thinner additions clustered around themselves and sometimes intersect while not following any pattern. We expected to see a decreasing trend on the required force with the increasing amounts of thinner, but this was not the case. Only 10% thinner made a significant decrease in the required force. Sudden jumps seen in **Supplementary Figure 1** occurred due to experimental setup dislocation.

4 Experimental setup for accumulated load calculation on syringe pumps

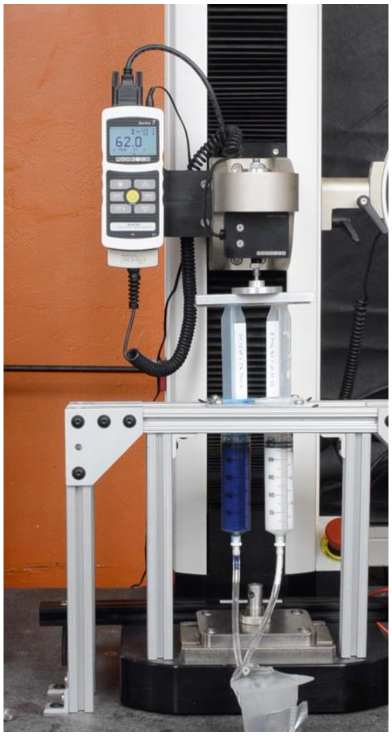

Supplementary Figure 2. Experimental setup for accumulated load calculation on syringe pumps.

The experimental apparatus consisted of the Mark-10 motorized tension/compression stand (ESM1500, Mark-10, NY), 1000N load cell (MR01-200-1, Mark-10, NY), and a custom built, aluminum, 60 ml, double syringe holder unit **(Supplementary Figure 2)**.

To reproduce the print flow speed, syringes were compressed at a speed of 0.64 mm/min. The selected tubing was abrasion-resistant polyurethane with an inner diameter of 1/8 inch and an outer diameter of 1/4 inch.

5 Full list of 3D print parameters

Supplementary Table 3. Full list of 3D print parameters used for the slicing program (Slic3r), the pumps, and the heaters.

	Parameter	Value		Parameter	Value
Layers and Perimeters	Layer height	0.8 mm	Extruder	Lift Z speed	40 mm/s
	First layer height	0.8 mm		Lift Z	1 mm
	Perimeters	0		Nozzle diameter	0.839 mm
	Solid layers top/bottom	5	Material	Material flow rate	24.7 ml/hr
	Avoid crossing perimeters	yes	Heaters	Heated bed temperature	45˚C
	Seam position	nearest		Convective heater temperature	80˚C
Infill	Infill density	100%		Convective heater fan speed	65%
	Fill pattern	rectilinear			
	Fill angle	45˚			
Speed	Print speed (speed of all print moves)	10 mm/s			
	Travel speed	50 mm/s			
Advanced	Default extrusion width	0.73 mm			
	Solid infill	125%			
	Infill perimeters overlap	0 mm			

6 Print limitations

Supplementary Table 4. List of design limitations and recommended parameters.

**Design Specs**	**Recommended Parameter**
Minimum overhang angle	Cannot be lower than 65° from level
Minimum supported wall thickness	1.2 mm
Maximum overhang gap	1 mm
Maximum horizontal bridge	5 mm
Minimum hollow circle diameter	3 mm

There are a couple of design limitations observed in robot fabrication. First, direct printing of the silicone elastomer without the addition of any support materials limits the achievable geometries. For example, the minimum overhang angle cannot be lower than 65° from level, which causes Pneu-net actuators to be hard to print. Second, due to the usage of the 3D printer slicing software optimized for thermoplastic material extrusion, generated print paths distort the printed parts at the sharp corners. A list of design limitations with the recommended parameters is available in **Supplementary Table 4**. Also, since the flow of the PDMS is continuous, unlike fused filament fabrication (FFF), a retraction command is not available to keep surface finish visually appealing. These limitations can be solved with the development of a new slicing software that can predict distortions and compensate them by encountering the properties of the viscoelastic materials and continuous flow.

7 Connecting the hexapus robot to the syringe pump before submerging

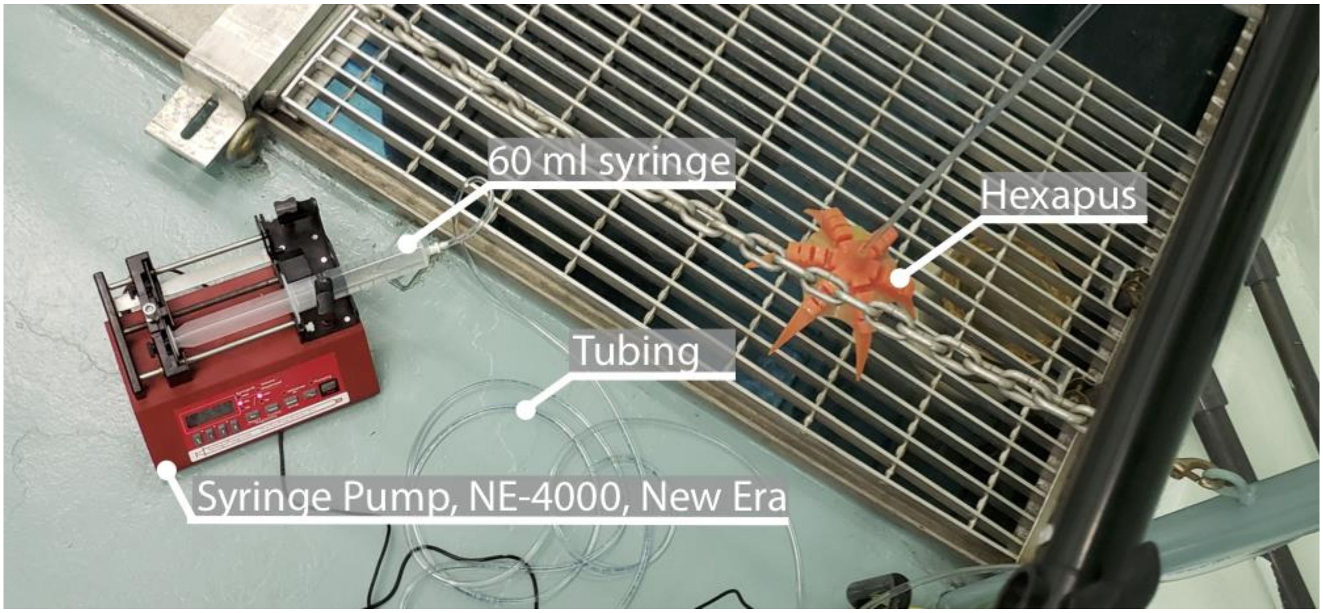

Supplementary Figure 3. Hexapus submerging setup.

Tap water was pumped and withdrawn in consecutive cycles into the hexapus by using a 60 ml syringe attached to a syringe pump (NE-4000, New Era, NY) at the max pump speed of 95.99 ml/min. The hexapus and the pump were connected by 6 meters of soft tubing with a 3.2 mm inner diameter. Before submerging, the internal channels of the hexapus were filled with tap water by squeezing the soft robot underwater. The tubing connected to the hexapus was not sealed.

8 Supplementary video 1: 3D printed hexapus robot and submerging process into the bulk shield tank.

Please see the attached video file in the downloads section of this journal page.

9 Measuring the underwater gamma irradiation rate

An underwater ion chamber shown in **Supplementary Figure 4** was used to measure the dose rate next to the hexapus robot inside the bulk shield tank.

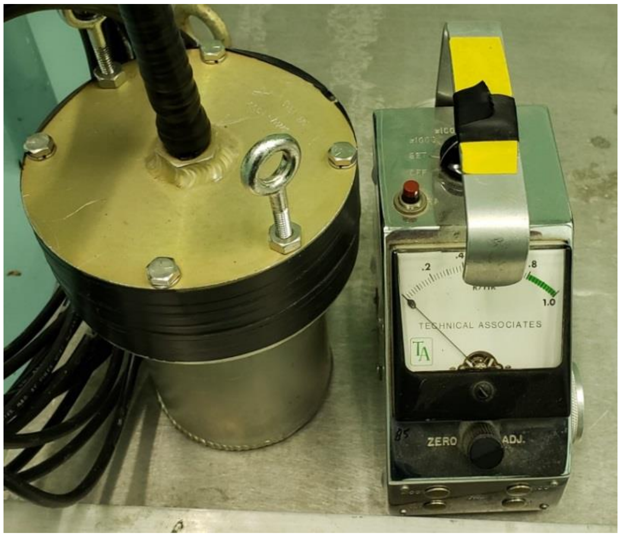

Supplementary Figure 4. An underwater ion chamber (CPMU Technical Associates, CA).

10 Robot design, selection, and fabrication

In this work, we manufactured three soft robots: a 4 channel tentacle (Marchese and Rus, 2016), a Pneu-net actuator (Mosadegh et al., 2014; Ilievski et al., 2011), and a soft hexapus robot (Frame et al., 2018). We selected our robot designs based on the most common (excluding the use of fabric) bending principles: eccentric void asymmetry (4 channel tentacle) and corrugated membrane asymmetry (Pneu-net actuator, hexapus) (Gorissen et al., 2017).

Supplementary Table 5. Differences at fabrication steps.

Fabrication Methods:	Lost Wax Casting (For 4 channel tentacle)	Lamination Casting (For Pneu-net actuator)	3D Printing (For each robot)
Step 1	Desired 3D CAD model preparation	Desired 3D CAD model preparation	3D computer-aided drawing (CAD) model preparation
Step 2	Inverting the CAD design for molding	Inverting the CAD design for molding	Turning a designed object into G-codes and running the print
Step 3	Designing the molds for lost wax casting	3D printing the molds	Installing and sealing the pneumatic actuation tubes
Step 4	3D printing the molds	Preparing the PDMS material	
Step 5	Preparing the PDMS material	Casting and de-gassing	
Step 6	Preparing the lost wax material	Curing inside an oven	
Step 7	Casting and de-gassing	De-molding and layer bonding	
Step 8	Curing inside an oven	Installing and sealing the pneumatic actuation tubes	
Step 9	De-molding and melting the wax material out		
Step 10	Installing and sealing the pneumatic actuation tubes		

11 Experimental setup for the performance comparison tests.

The experimental setup is shown in **Supplementary Figure 5** consists of 4 main components: 1) a high precision syringe pump (PHD ULTRA, Harvard Apparatus, MA) with two 60 ml syringes attached (BD Plastic, NJ) to inject air at a rate of 2 ml/s, 2) a pressure gauge (Media Gauge, SSI Technologies, WI), 3) a force indicator (M7i, Mark-10, NY) with an attached load cell (MR03-5-1, Mark-10, NY), and 4) a 3D printed experiment platform with specific clamps for each robot. With this setup[8], we controlled the volume of the air flow, measured the pressure accumulated inside the actuators, and measured the resulting output force. Blocked force experiments were repeated at different heights (5 mm, 10 mm, and 15 mm). The same setup is used with components 1, 2, a digital video camera and an image editing software for the bend angle experiments.

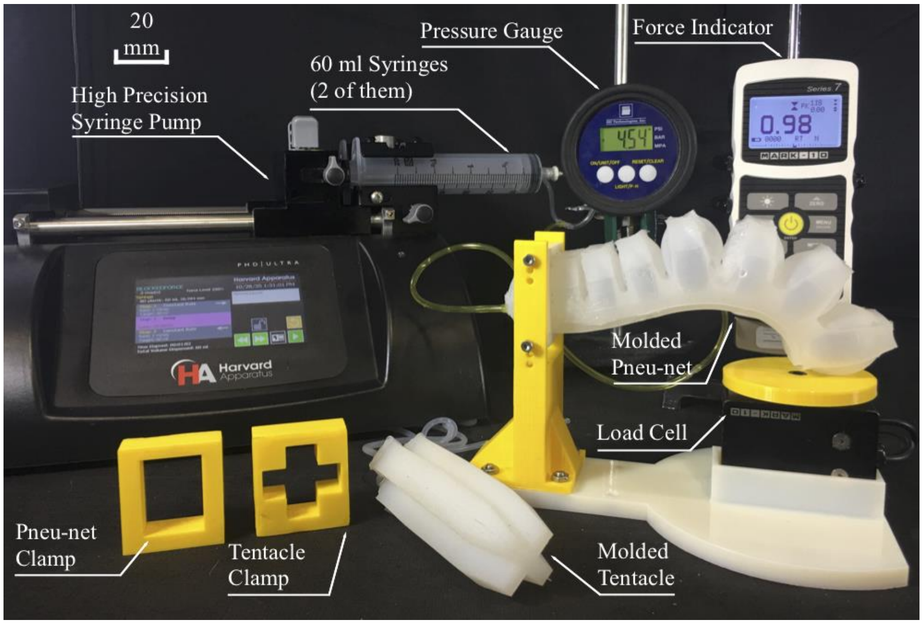

Supplementary Figure 5. Experimental setup for the blocked force measurements (Yirmibesoglu et al., 2018).

[8] Experimental setup design files: https://www.thingiverse.com/thing:3200886 (Accessed: January 2019)

12 100% tensile modulus line fit into experimental data collected from dumbbell test samples

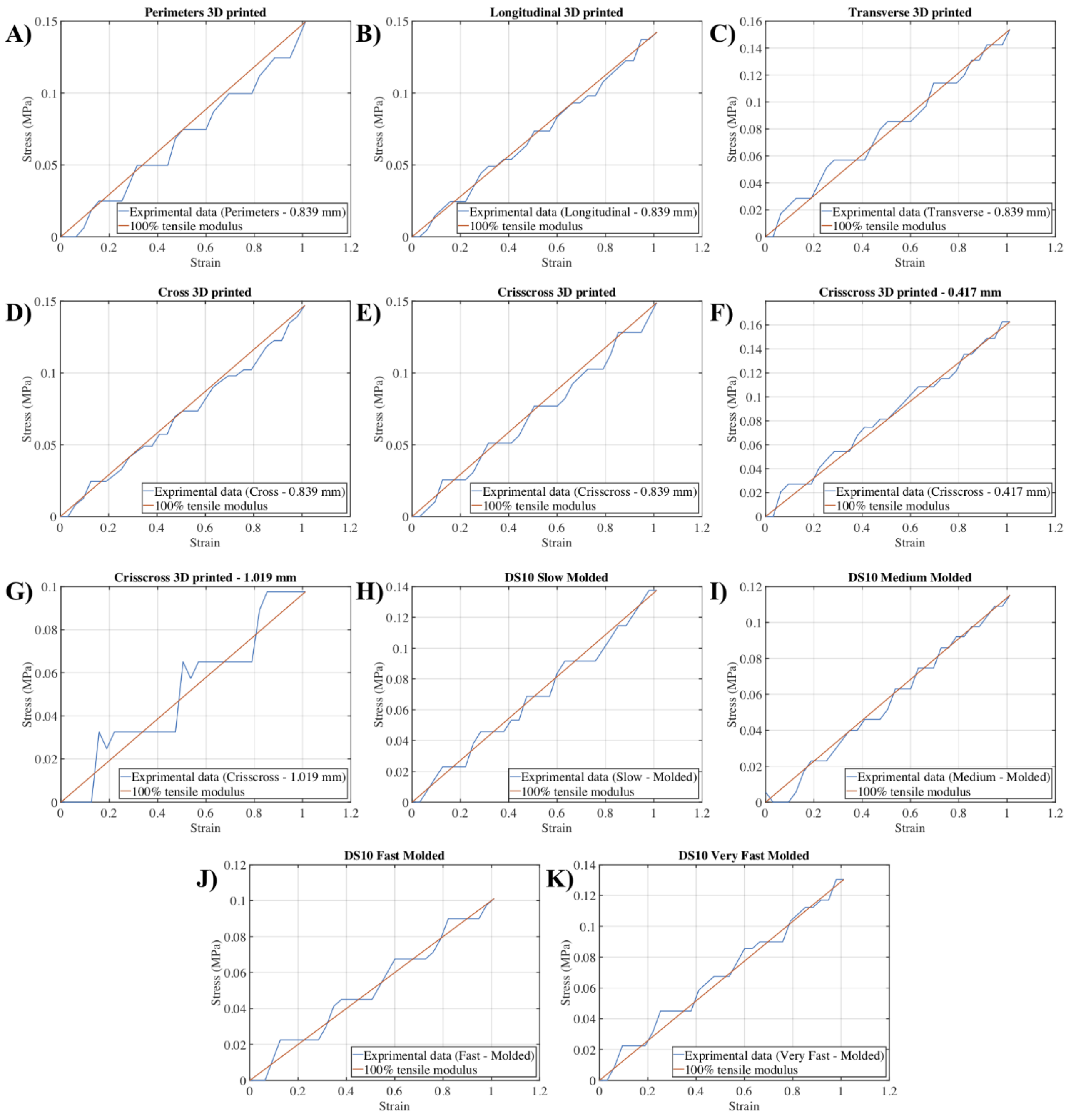

Supplementary Figure 6. 100% tensile modulus line fit into experimental data collected from dumbbell test samples. **A)** Perimeters 3D printed, **B)** Longitudinal 3D printed, **C)** Transverse 3D printed, **D)** Cross 3D printed, **E)** Crisscross 3D printed 0.839 mm nozzle, **F)** Crisscross 3D printed with 0.417 mm nozzle, **G)** Crisscross 3D printed with 1.019 mm nozzle, **H)** Molded with DS10-slow, **I)** Molded with DS10-medium, **J)** Molded with DS10-fast, **K)** Molded with DS10-very-fast.

In **Supplementary Figure 6,** we fit a 100% tensile modulus line into our experimental data for each dumbbell test category. Due to sensor resolution, a systematic error of ±0.5N was introduced into all test results. Experimental data shown in the **Supplementary Figure 6** is an average value of all the samples (minimum of n=3, maximum of n=6) in that category. The Young's modulus is the slope of the line fit.

13 Blocked force comparisons at different heights

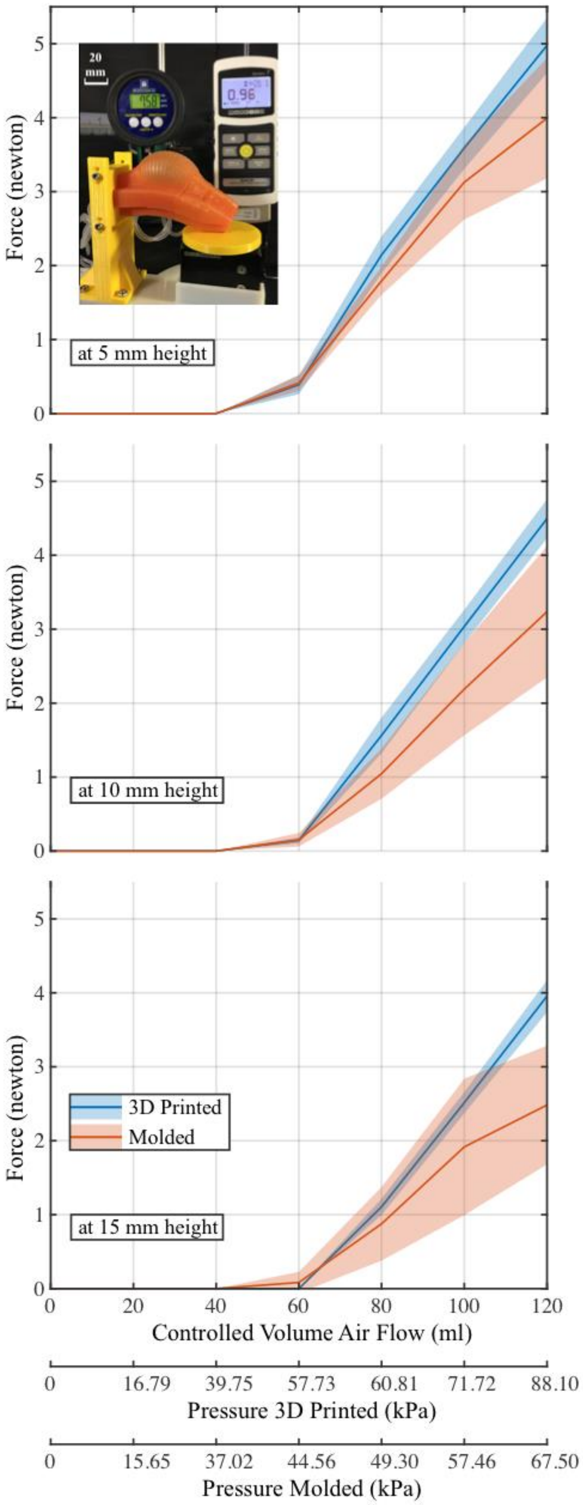

Supplementary Figure 7. Blocked force experiments at different tentacle attachment heights above the sensor surface.

14 Uneven channel geometry caused by bending of the channels due to lost wax cast cores

For proper soft actuator operation, actuation channels should be consistent and maintain constant wall thickness. In the lost wax casting technique, due to soft wax cores and manual handling, the wax cores would occasionally bend as seen in **Supplementary Figure 8**. This bend creates uneven wall thicknesses d2 > d1, resulting in different performance between fabricated actuators. A channel can explode due to a thin wall or cannot bend in the desired angle due to a thick wall.

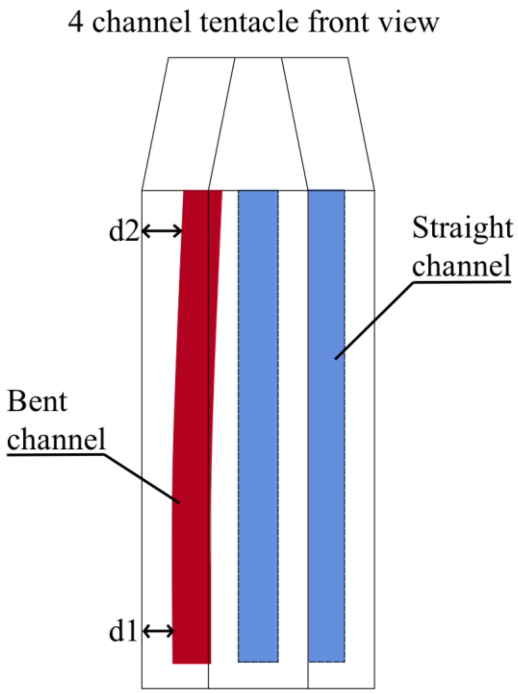

Supplementary Figure 8. The effect of using lost wax cast cores in the fabrication of long, internal channels.

15 Weight comparison between fabricated dumbbell test samples

Supplementary Table 6. Weight measurements of the 3D printed dumbbell test samples used for calculating Young's modulus.

3D print direction	Sample 1	Sample 2	Sample 3	Sample 4	Sample 5	Sample 6
Perimeters	5.24 g	5.19 g	5.31 g	5.23 g	-	-
Longitudinal	5.87 g	5.8 g	5.9 g	5.93 g	5.81 g	-
Transverse	5.03 g	5.12 g	5.11 g	5.10 g	5.10 g	-
Cross	5.63 g	5.56 g	5.52 g	5.66 g	5.63 g	5.67 g
Crisscross (0.417 mm)	5.4 g	5.3 g	5.43 g	5.38 g	5.4 g	-
Crisscross (0.839 mm)	5.01 g	5.24 g	5.20 g	-	-	-
Crisscross (1.019 mm)	4.5 g	4.48 g	4.51 g	4.51 g	-	-

Supplementary Table 7. Weight measurements of the molded dumbbell test samples used for calculating Young's modulus.

Dragon skin 10 product types	Sample 1	Sample 2	Sample 3	Sample 4	Sample 5	Sample 6
Slow (molded)	5. 54 g	5.58 g	5.78 g	-	-	-
Medium (molded)	5.63 g	5.50 g	5.57 g	5.60 g	-	-
Fast (molded)	5.71 g	5.73 g	5.72 g	5.75 g	5.54 g	5.68 g
Very fast (molded)	5.77 g	5.80 g	5.79 g	5.83 g	5.66 g	-

We also recorded the weights of each dumbbell test samples to make sure that there was not an outlier in each category. Any samples that deviated more than 0.5 g from the average in each category were eliminated. Depending on the print direction and nozzle size we observed differences in average weight between each category shown in **Supplementary Table 6.** We also recorded the weights of molded dumbbell test pieces shown in **Supplementary Table 7.** When both tables are compared, we observed that molded samples are heavier than most of the 3D printed sample categories. Further experiments are required to investigate the role of weight in the stiffness analysis of PDMS material.

16 Effect of print direction on the surface finish

Due to the continuous flow of the silicone material during the print process, the print direction has a significant effect on the surface finish of the material. If the print direction causes the print path to move between print islands, the nozzle starts to drag materials. As a result, extruder marks occur on the printed object's surface. We observed these marks mostly on longitudinal, perimeter and cross print directions shown in **Supplementary Figure 9**. The most successful print directions, which gave us high-quality surface finish as seen in the molded samples, were crisscross and transverse.

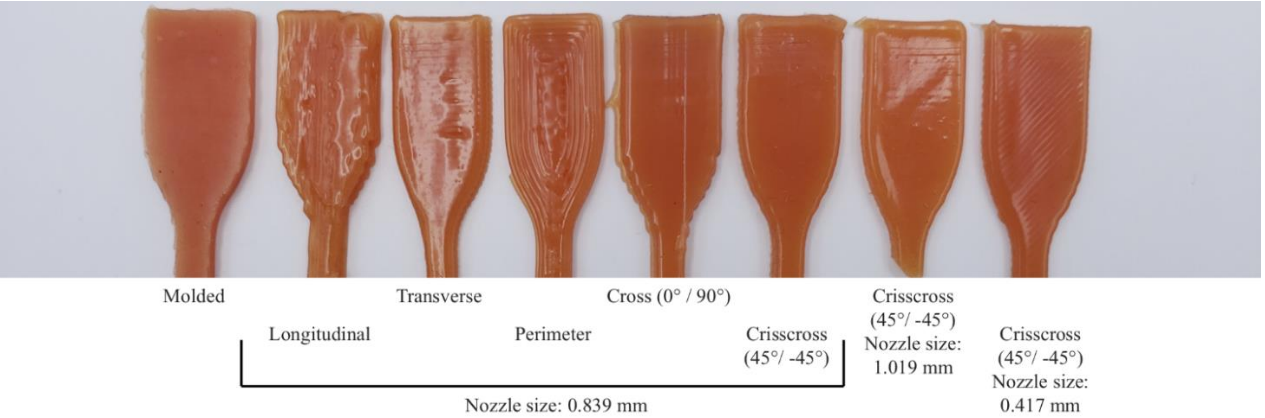

Supplementary Figure 9. Effect of print direction on the surface finish. For comparison, molded and multiple nozzle sizes are also included.

A potential solution to avoid dragging the nozzle through the uncured material is to lift the nozzle away from the part as it travels. However, the flow of the PDMS is continuous, unlike fused filament fabrication (FFF), and a retraction command is not available to keep surface finish visually appealing. This result means, if the extruder travels over the printed surface at the same layer height twice or more, the problems such as over-extrusion and material dragging will cause impurities at the surface as seen in the **Supplementary Figure 9** perimeter, and longitudinal print directions.

17 Effects of print direction on Young's modulus

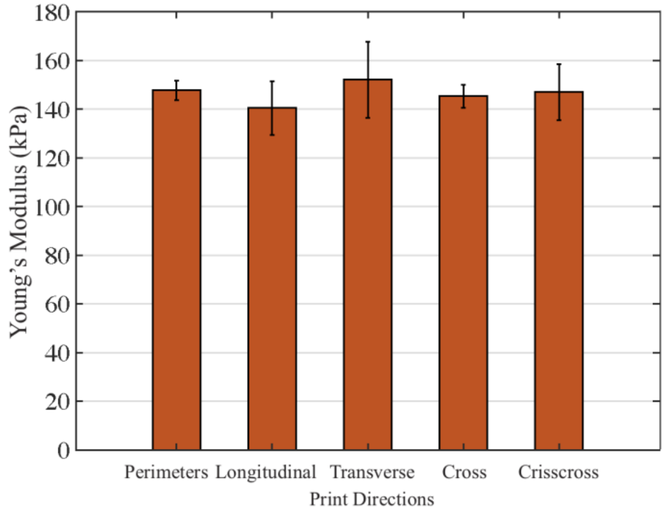

Supplementary Figure 10. Effects of print direction on Young's modulus. (Fixed nozzle size: 0.839 mm). Graphical representation of **Table 3**.

18 References

Frame, Jennifer, Nick Lopez, Oscar Curet, and Erik D Engeberg. 2018. "Thrust Force Characterization of Free-Swimming Soft Robotic Jellyfish." *Bioinspiration & Biomimetics* 13 (6): 064001. https://doi.org/10.1088/1748-3190/aadcb3.

Gorissen, Benjamin, Dominiek Reynaerts, Satoshi Konishi, Kazuhiro Yoshida, Joon-Wan Kim, and Michael De Volder. 2017. "Elastic Inflatable Actuators for Soft Robotic Applications." *Advanced Materials* 29 (43): 1604977. https://doi.org/10.1002/adma.201604977.

Ilievski, Filip, Aaron D. Mazzeo, Robert F. Shepherd, Xin Chen, and George M. Whitesides. 2011. "Soft Robotics for Chemists." *Angewandte Chemie International Edition* 50 (8): 1890–95. https://doi.org/10.1002/anie.201006464.

Marchese, Andrew D., and Daniela Rus. 2016. "Design, Kinematics, and Control of a Soft Spatial Fluidic Elastomer Manipulator." *The International Journal of Robotics Research* 35 (7): 840–69. https://doi.org/10.1177/0278364915587925.

Mosadegh, Bobak, Panagiotis Polygerinos, Christoph Keplinger, Sophia Wennstedt, Robert F. Shepherd, Unmukt Gupta, Jongmin Shim, Katia Bertoldi, Conor J. Walsh, and George M. Whitesides. 2014. "Pneumatic Networks for Soft Robotics That Actuate Rapidly." *Advanced Functional Materials* 24 (15): 2163–70. https://doi.org/10.1002/adfm.201303288.

Ober, Thomas J, Daniele Foresti, and Jennifer A Lewis. 2015. "Active Mixing of Complex Fluids at the Microscale." *Proceedings of the National Academy of Sciences of the United States of America* 112 (40): 12293–98. https://doi.org/10.1073/pnas.1509224112.

Yirmibesoglu, Osman Dogan, John Morrow, Steph Walker, Walker Gosrich, Reece Canizares, Hansung Kim, Uranbileg Daalkhaijav, Chloe Fleming, Callie Branyan, and Yigit Menguc. 2018. "Direct 3D Printing of Silicone Elastomer Soft Robots and Their Performance Comparison with Molded Counterparts." In *2018 IEEE International Conference on Soft Robotics (RoboSoft),* 295–302. Livorno: IEEE. https://doi.org/10.1109/ROBOSOFT.2018.8404935.
